# Estimated Costs and Outcomes Associated With Use and Nonuse of Medications for Opioid Use Disorder During Incarceration and at Release in Massachusetts

**DOI:** 10.1001/jamanetworkopen.2023.7036

**Published:** 2023-04-14

**Authors:** Avik Chatterjee, Michelle Weitz, Alexandra Savinkina, Alexandria Macmadu, R. W. M. A. Madushani, Ruth A. Potee, Danielle Ryan, Sean M. Murphy, Alexander Y. Walley, Benjamin P. Linas

**Affiliations:** 1 Grayken Center for Addiction, Clinical Addiction Research and Education Unit, Section of General Internal Medicine, Department of Medicine, Boston Medical Center and Boston University Chobanian & Avedisian School of Medicine, Boston, Massachusetts; 2 Section of Infectious Diseases, Department of Medicine, Boston Medical Center and Boston University Chobanian & Avedisian School of Medicine, Boston, Massachusetts; 3 Epidemiology of Microbial Diseases, Yale School of Public Health, New Haven, Connecticut; 4 Department of Epidemiology, Brown University School of Public Health, Providence, Rhode Island; 5 Franklin County House of Corrections, Greenfield, Massachusetts; 6 Department of Population Health Sciences, Weill Cornell Medical College, New York, New York

## Abstract

**Importance:**

Most prisons and jails in the US discontinue medications for opioid use disorder (MOUD) upon incarceration and do not initiate MOUD prior to release.

**Objective:**

To model the association of MOUD access during incarceration and at release with population-level overdose mortality and OUD-related treatment costs in Massachusetts.

**Design, Setting, and Participants:**

This economic evaluation used simulation modeling and cost-effectiveness with costs and quality-adjusted life-years (QALYs) discounted at 3% to compare MOUD treatment strategies in a corrections cohort and an open cohort representing individuals with OUD in Massachusetts. Data were analyzed between July 1, 2021, and September 30, 2022.

**Exposures:**

Three strategies were compared: (1) no MOUD provided during incarceration or at release, (2) extended-release (XR) naltrexone offered only at release from incarceration, and (3) all 3 MOUDs (naltrexone, buprenorphine, and methadone) offered at intake.

**Main Outcomes and Measures:**

Treatment starts and retention, fatal overdoses, life-years and QALYs, costs, and incremental cost-effectiveness ratios (ICERs).

**Results:**

Among 30 000 simulated incarcerated individuals with OUD, offering no MOUD was associated with 40 927 (95% uncertainty interval [UI], 39 001-42 082) MOUD treatment starts over a 5-year period and 1259 (95% UI, 1130-1323) overdose deaths after 5 years. Over 5 years, offering XR-naltrexone at release led to 10 466 (95% UI, 8515-12 201) additional treatment starts, 40 (95% UI, 16-50) fewer overdose deaths, and 0.08 (95% UI, 0.05-0.11) QALYs gained per person, at an incremental cost of $2723 (95% UI, $141-$5244) per person. In comparison, offering all 3 MOUDs at intake led to 11 923 (95% UI, 10 861-12 911) additional treatment starts, compared with offering no MOUD, 83 (95% UI, 72-91) fewer overdose deaths, and 0.12 (95% UI, 0.10-0.17) QALYs per person gained, at an incremental cost of $852 (95% UI, $14-$1703) per person. Thus, XR-naltrexone only was a dominated strategy (both less effective and more costly) and the ICER of all 3 MOUDs compared with no MOUD was $7252 (95% UI, $140-$10 018) per QALY. Among everyone with OUD in Massachusetts, XR-naltrexone only averted 95 overdose deaths over 5 years (95% UI, 85-169)—a 0.9% decrease in state-level overdose mortality—while the all-MOUD strategy averted 192 overdose deaths (95% UI, 156-200)—a 1.8% decrease.

**Conclusions and Relevance:**

The findings of this simulation-modeling economic study suggest that offering any MOUD to incarcerated individuals with OUD would prevent overdose deaths and that offering all 3 MOUDs would prevent more deaths and save money compared with an XR-naltrexone–only strategy.

## Key Points

**Question:**

Is provision of medications for opioid use disorder (MOUD) during incarceration associated with fewer overdose deaths?

**Findings:**

This economic evaluation of a model of the natural history of OUD in Massachusetts found that a strategy offering buprenorphine, methadone, and naltrexone during incarceration was associated with 192 fewer overdose deaths (a 1.8% reduction) and was less costly than a naltrexone-only strategy averting 95 overdose deaths (a 0.9% reduction). The 3-MOUD strategy was also cost-effective at $7252 per quality-adjusted life-year gained.

**Meaning:**

These findings suggest that offering 3 MOUDs during incarceration is a life-saving, cost-effective intervention.

## Teaser

This economic evaluation estimates the cost-effectiveness and overdose mortality rates associated with a Massachusetts policy requiring that all incarcerated individuals be screened for opioid use disorder (OUD) and offered all US Food and Drug Administration–approved medications for OUD treatment.

## Introduction

Overdose is the leading cause of accidental death in the US.^1^ Medications for opioid use disorder (MOUD) are lifesaving, but only a fraction of people with OUD initiate MOUD treatment.^2^ A substantial barrier to initiating MOUD treatment, particularly agonist medications such as methadone hydrochloride or buprenorphine hydrochloride, is patient concern about experiencing withdrawal symptoms due to forced cessation of treatment.^3,4^ Patients commonly experience withdrawal in jails and prisons because most carceral facilities discontinue agonist medications and do not work to initiate MOUD and link people to care at release.^5^ This prevailing policy is associated with a 12.7-fold higher rate of death among people leaving correctional facilities.^6^

In the face of increasing overdose deaths and elevated risk post incarceration, some carceral systems have implemented programs to continue treatment with MOUD during incarceration and to routinely screen for OUD and link patients to MOUD providers on release. Empirical studies demonstrate that individuals participating in MOUD programs while incarcerated are more likely to be retained in treatment post release,^7,8,9,10,11^ that they report a reduction in opioid use,^7,8^ and that such programs are likely cost-effective.^12^ As stated in the National Academies of Sciences, Engineering, and Medicine’s 2019 consensus report: “To stem the opioid crisis, it is critical for all FDA [US Food and Drug Administration]–approved options to be available for all people with OUD.”^13^

In April 2022, the Massachusetts US Attorney’s office entered into agreements with all federal, state, and county correctional facilities to continue an individual’s prescribed, FDA-approved MOUD on incarceration in Massachusetts. This agreement does not require screening and MOUD treatment initiation, if indicated, though several facilities plan to implement such programs. We therefore used the Researching Effective Strategies to Prevent Opioid Death (RESPOND) simulation model to assess the association that a policy requiring all Massachusetts jails and prisons to screen for OUD and offer all FDA-approved MOUD during incarceration would have with overdose mortality and cost from a health care perspective and a modified societal perspective.^14^

## Methods

### RESPOND Model Structure

This study followed the Consolidated Health Economic Evaluation Reporting Standards (CHEERS) reporting guideline. We utilized RESPOND, a dynamic population, state-transition simulation model of OUD in Massachusetts, to project outcomes, costs, and cost-effectiveness of various strategies for initiating MOUD treatment during incarceration. We conducted analyses between July 1, 2021, and September 30, 2022. The RESPOND model, which is described in detail in previous work^15^ and in the eAppendix in Supplement 1,^16^ simulates the lifetime progression of a state-level population living with OUD as a series of transitions between health states. The model can simulate a closed cohort of people with OUD as it progresses over a lifetime (similar to a traditional longitudinal cohort study), or it can simulate a dynamic population of people with OUD, to allow for changes in state-level OUD rates, both among existing residents as well as those moving into Massachusetts (similar to following the entire population of people living with OUD in the state). The RESPOND model project was reviewed by the Boston University Medical Campus and Boston Medical Center Institutional Review Board and was deemed not to be human participant research.

The model characterizes the relapsing-remitting nature of OUD with 4 health states representing permutations of active vs nonactive and noninjection vs injection drug use (eFigures 1 and 2 and eTable 1 in Supplement 1). Active use states are characterized by a lower quality of life and greater use of health care services than nonactive states. Active use states also carry a risk of overdose, which is highest with injection use. Among those who overdose, a proportion experiences a fatal overdose (eTables 2-5 in Supplement 1). Those who survive an overdose continue in the simulation. We simulate age- and sex-adjusted competing risks of death from drug use–associated conditions other than overdose using standardized mortality ratios (eTables 6 and 7 in Supplement 1).^17^

Throughout the simulation, the population faces an incarceration risk that is higher when actively using drugs, and even more so when injecting drugs. Thus, individuals can be incarcerated more than once over their lifetime. The demographics of the incarcerated population are determined by the combination of the relative rates. During incarceration, we assumed the potential for ongoing drug use with risk of overdose mortality.^18^ Following release from correctional settings without linkage to MOUD, there is a 4-week posttreatment state that simulates the heightened risk of overdose in the weeks following release from incarceration.^6^

Throughout the simulation, the population that is actively using opioids may seek MOUD treatment in the community, including outpatient buprenorphine or methadone at opioid treatment programs (eFigure 2 and eTable 8 in Supplement 1). During the course of outpatient MOUD treatment, there is bidirectional movement between active and nonactive drug-use states, with the balance of that movement tending to favor the nonactive state (eTables 9 and 10 and eFigure 3 in Supplement 1).^19^ In addition, each MOUD has an independent association with the overdose rate.^20,21^ In every 2-week time step of the simulation, the population engaged with MOUD faces a probability of disengaging from care (eTable 11 in Supplement 1).

### Data and Parameter Estimation

Table 1 provides details of parameter values and uncertainty intervals (UIs) for this analysis.^9,10,11,22,23,24,25,26,27,28,29,30,31,32,33,34,35,36,37,38,39,40,41,42,43^ Although we provide parameter values in monthly rates, we converted monthly rates to weekly probabilities for use in the model.

**Table 1.** RESPOND Parameters

RESPOND parameter	Baseline value (range)	PSA distribution	Source
**Population demographics and epidemiology**			
Proportion male	0.68 (0.54-0.72)	β^a^	Massachusetts Department of Public Health,^22^ 2022
Mean age, y	37 (27-47)	β^a^	Massachusetts Department of Public Health,^22^ 2022
Proportion with injection drug use at baseline	0.25 (0.20-0.30)	NA	MA PHD,^23^ 2018
Proportion actively using at baseline	0.84 (0.67-1.00)	NA	MA PHD,^23^ 2018
Standardized mortality ratio for drug use			
Injection drug users	5.1 (4.4-5.7)	γ	2010 Census^24^; MA PHD,^23^ 2018
Noninjection drug users	2.1 (1.8-2.3)	γ	2010 Census^24^; MA PHD,^23^ 2018
Movement into corrections, monthly rate per 1000 people	7.8 (1.2-26.4)	Uniform	Bertram and Jones,^25^ 2019; Cannata et al,^26^ 2019
Mean corrections length of stay, wk	4 (2-6)	Uniform	Zeng,^27^ 2019
Linkage to MOUD after corrections, %			
Buprenorphine	48 (43-53)	Uniform	Gordon, et al,^9^ 2014; Magura et al,^10^ 2019; Gordon et al,^28^ 2017
Methadone	82 (74-90)	Uniform	Magura et al,^10^ 2019; Rich et al,^11^ 2015
Naltrexone	45 (40-78)	Uniform	Jarvis et al,^29^ 2018; Friedmann et al,^30^ 2018
Movement to MOUD treatment, monthly rate per 1000 people	4.6 (0.6-13.2)	NA	Bronson et al,^31^ 2017; Barocas et al,^32^ 2018
Buprenorphine	19.9 (11.4-41.8)	β	MA PHD,^23^ 2018
Methadone	5.9 (2.4-24.4)	β	MA PHD,^23^ 2018
Naltrexone	2.3 (0.7-21.5)	β	MA PHD,^23^ 2018
Proportion retained in MOUD treatment at 6 mo			
Buprenorphine	0.34 (0.17-0.51)	β	Morgan et a,l^33^ 2018
Methadone	0.55 (0.28-0.84)	β	Soyka et al,^34^ 2008
Naltrexone	0.21 (0.11-0.32)	β	Morgan et al,^33^ 2018
Overdose monthly rate per 1000 people			
No treatment	6.75 (6.00-11.20)	Uniform	MA PHD,^23^ 2018
Buprenorphine	2.73 (2.20-4.40)	Log normal^b^	Morgan et al,^20^ 2019
Naltrexone	5.83 (4.80-9.20)	Log normal^b^	Morgan, et al,^20^ 2019
Methadone	5.07 (4.40-8.40)	Log normal^b^	Sordo et al,^21^ 2017
Rate per 100 000 in correctional facilities	3.46 (3.00-5.60)	Uniform^b^	Carson,^35^ 2021
Posttreatment	12.95 (11.40-21.60)	Uniform	NA
Fatal overdose proportion	0.14 (0.13-0.15)	β	MA PHD,^23^ 2018
**Costs (health care sector)**			
Weekly treatment costs (visit and medication), $			
Detox	2863 (805-8204)	Normal	Evans et al,^36^ 2022; McCollister et al,^37^ 2019
Buprenorphine hydrochloride (16 mg/d)	114 (75-163)	Normal	US Department of Veterans Affairs,^38^ 2023
Methadone hydrochloride (80 mg/d)	126 (116-136)	Normal	National Institute on Drug Abuse,^39^ 2021
Naltrexone (380 mg/mo)	327 (316-338)	Normal	US Department of Veterans Affairs,^38^ 2023
Corrections buprenorphine (16 mg/d)	115 (92-138)	Normal	Mace et al,^40^ 2022
Corrections methadone (80 mg/d)	126 (116-136)	Normal	National Institute on Drug Abuse,^39^ 2021
Corrections naltrexone (1 injection/mo)	250 (200-250)	Normal	Mace et al,^40^ 2022
Overdose costs, $			
Fatal overdose	858 (443-1329)	Uniform	Coffin et al,^41^ 2013; Jiang et al,^42^ 2017
Nonfatal overdose	4557 (2279-6836)	Uniform	Coffin et al,^41^ 2013; Jiang et al,^42^ 2017
Weekly incarceration	840	NA	*Federal Register*,^43^ 2021

Abbreviations: MA PHD, Massachusetts Public Health Data Warehouse; MOUD, medication for opioid use disorder; NA, not applicable; PSA, probabilistic sensitivity analysis; RESPOND, Researching Effective Strategies to Prevent Opioid Death.

^a^ Modeled as age- and sex-stratified proportion of total cohort.

^b^ Modeled as a multiplier to the no treatment overdose rate.

The primary data source for demographic characteristics and treatment-seeking parameters in RESPOND is the Massachusetts Public Health Data Warehouse (MA PHD),^23^ a longitudinally linked administrative records database that includes service encounter data from more than 16 statewide agencies. The work performed by the Massachusetts Department of Public Health was mandated by law and performed by a public health authority. The Massachusetts Department of Public Health was not engaged in human participant research, and no institutional review board approval was required. To estimate the size of the population with OUD, we used capture-recapture analysis of MA PHD data sets (eTable 12 and eFigure 7 in Supplement 1).^32^ To estimate the yearly entering cohort, rates of seeking MOUD treatment, and incarceration rates, we tabulated admissions observed in MA PHD (eTables 13 and 14 in Supplement 1).^26^

Data for the natural history of OUD come from cohort studies in the medical literature (Table 1).^9,10,11,20,21,22,23,24,25,26,27,28,29,30,31,32,33,34,35,36,37,38,39,41,42,43^ Data on overdose mortality during incarceration are limited^18^; however, based on a federal report, we estimated overdose mortality rates during incarceration to be half of that in the general population.^35^ We estimated parameters on the efficacy of MOUD for treatment of opioid use and mortality using toxicology data from the National Institute on Drug Abuse Clinical Trials Network (eAppendix in Supplement 1) and the medical literature. We used clinical trial data to estimate the pharmacological efficacy of MOUD among those who remain engaged with treatment, but not to estimate clinical retention in care. We analyzed clinical claims data to estimate retention and loss to follow-up during MOUD treatment.

### Costs

We conducted the base-case analysis and sensitivity analyses from a health care sector perspective. We also provide budgetary impact results from a modified societal perspective that include the cost of incarceration. We denominated costs in 2019 US dollars and discounted 3% annually.^44^ RESPOND includes 3 cost components: (1) costs of all health care services other than OUD treatment, (2) costs of OUD treatment, and (3) costs related to opioid overdose. We estimated the cost of all 3 components using econometric multivariable models and person-level data from the National Institute on Drug Abuse Clinical Trials Network trial 0051 (eTables 15-19 in Supplement 1).^37,45^

### Health State Utilities

We used published estimates of health state utilities among persons who use drugs.^46^ All quality-of-life measures are health state utilities collected using the standard gamble method.^47^ We estimated multistate utility functions using the minimal utility approach.^44^ We used the multiplicative utility approach in sensitivity analyses.

### Statistical Analysis

We used R, version 4.0.5 (R Project for Statistical Computing) for all analyses. First, we used RESPOND to model a closed cohort of 30 000 incarcerated individuals in Massachusetts. This analysis simulates the experience of a study in which all members of a cohort are exposed to the intervention and followed up over a lifetime of tracking outcomes. We report both 5-year and lifetime outcomes from this closed cohort. Next, we repeated the analysis, but this time using RESPOND to simulate an open cohort representing the population of Massachusetts. In the open cohort analysis, rates of incarceration were a function of drug use status, with active use state having higher rates than nonactive state and injection drug use having higher rates than noninjection drug use. This analysis simulates the effect of a change in carceral policy on treating OUD behind bars on state-level overdose outcomes. We ran the open cohort simulation over 8 years prior to any intervention (2013-2020) and then for 5 years of intervention time (2021-2025).

For both the closed and open cohort approaches, we modeled 3 strategies:

1. No MOUD was provided during incarceration.
2. Extended-release (XR) naltrexone only was offered on release from incarceration, currently the most common approach in carceral settings that offer any OUD treatment. We estimated that 66% of those offered XR-naltraxone on release from jail would accept it, based on existing empirical data from ongoing pilot studies in which 50% to 82% uptake has been observed,^36^ and that 45%^29^ would then link to care in the community.
3. All 3 MOUDs were offered at intake to jail, again with an uptake of 66% (an all-MOUD strategy). Of those initiating MOUD, we estimated that 76% would start buprenorphine therapy, 23% would start methadone therapy, and 1% would start XR-naltrexone therapy,^36^ based on existing empirical data.^48^ Linkage after corrections was assumed to be 48% for buprenorphine, 82% for methadone, and 45% for XR-naltrexone based on existing studies.^9,10,11,28,29^

#### Incremental Cost-effectiveness Ratios and 5-Year Budgetary Impact

We calculated incremental cost-effectiveness ratios (ICERs) using output from the lifetime closed cohort simulation. We calculated ICERs by dividing the incremental mean discounted lifetime cost between a strategy and the next less costly strategy by the incremental discounted quality-adjusted life-years (QALYs) gained between the strategies. We considered any strategy that provided worse outcomes at a higher mean lifetime cost than another strategy option as well as those that provided worse outcomes at a higher cost per discounted QALY gained to be dominated strategies and eliminated them from the ICER calculations.^44^ We used the open cohort simulation to project budgetary impact to Massachusetts over a 5-year horizon, reporting budgetary impact in undiscounted US dollars from a health care sector and modified societal perspective.

#### Sensitivity Analysis

We performed a series of deterministic sensitivity analyses, varying key parameters one at a time to the upper and lower bounds of their feasible ranges (Table 1) to identify important drivers of the results and characterize threshold values that could result in a different conclusion. Sensitivity analyses of special interest included percentage initiation of XR-naltrexone in the XR-naltrexone–only strategy to find the percentage uptake at which overdose deaths averted were comparable between the XR-naltrexone strategy and an all-MOUD strategy; the rate of uptake to MOUD; and rates of linkage to community-based MOUD after release from jail.

We also performed probabilistic sensitivity analyses in which we defined probability distribution functions for each parameter and ran the model 1000 times, with each run using a different set of randomly chosen parameter values. We report UIs as the 2.5th and 97.5th percentiles of the empirically observed distribution from the probabilistic sensitivity analysis. A cost-effectiveness acceptability curve is shown in eFigure 6 in Supplement 1.

## Results

### Closed Cohort of Incarcerated Individuals

**Figure 1.**
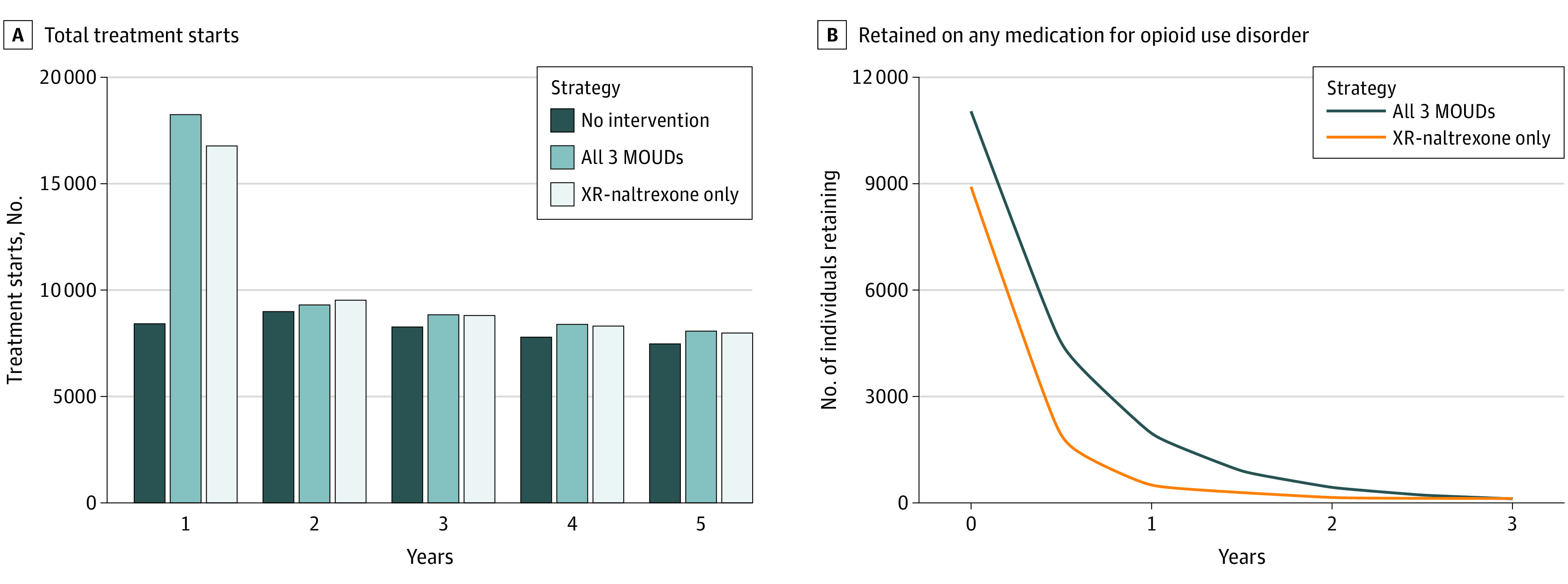
Total Treatment Starts and Treatment Retention Among Incerated Individuals With Opioid Use Disorder Results of a simulation analysis of various strategies for treating opioid use disorder in carceral settings. A, The vertical axis is denominated in terms of total treatment starts over the lifetime of the cohort. Each cluster of bars represents the year of the simulation. Each color bar represents a different strategy. B, Visualization of the number of people retained with any medication for opioid use disorder (MOUD) for both the extended-release (XR) naltrexone–only strategy, and for the all-MOUD strategy.

In the simulated model of 30 000 incarcerated individuals, without any MOUD treatment offered in correctional settings, there were 40 927 (95% UI, 39 001-42 082) MOUD treatment initiations over 5 years, representing treatment starts in the community after release. Routinely offering XR-naltrexone at release from jail or prison resulted in an additional 10 466 MOUD treatment initiations (95% UI, 8515-12 201), mostly XR-naltrexone, over the 5-year time horizon, and an additional 20 639 (95% UI, 10 724-30 760) MOUD treatment initiations over a lifetime (Figure 1A and eTable 20 in Supplement 1). In comparison, the all-MOUD strategy resulted in an additional 11 923 MOUD treatment starts (95% UI, 10 861-12 911) over the 5-year time horizon, and 24 131 MOUD treatment initiations over a lifetime, when compared with the status quo. The difference in treatment starts was predominantly driven by increases in initiations of methadone and buprenorphine treatment.

Treatment retention was higher under the all-MOUD strategy (Figure 1B and eTable 21 in Supplement 1). At 6 months after release, assuming the all-MOUD strategy, 41% of those taking MOUD upon release with linkage to community-based treatment were still receiving MOUD, vs 21% of those under the XR-naltrexone–only strategy.

Assuming no MOUD treatment initiations in corrections, the model estimated 296 (95% UI, 274-305) deaths due to overdose at 1 year and 1259 (95% UI, 1130-1323) deaths due to overdose at 5 years. Compared with no MOUD, each of the intervention strategies resulted in fewer deaths. In the XR-naltrexone–only strategy, 25 (95% UI, 21-52) deaths were averted at 1 year, an 8.4% decrease; 40 (95% UI, 16-50) deaths were averted over the 5-year period, a 3.2% decrease; and 0.08 (95% UI, 0.05-0.11) QALYs were gained per person. In the all-MOUD strategy, 51 (95% UI, 30-70) deaths were averted at 1 year, a decrease of 17.2%, and a total of 83 (95% UI, 72-91) deaths were averted over the 5-year period, a 6.6% decrease (Table 2 and eFigure 4 in Supplement 1).

**Table 2.** Overdose Deaths in a Simulated Closed Cohort and Open Cohort Massachusetts Population

Intervention	No. of overdose deaths (95% UI) by study year						Difference compared with no intervention	Difference compared with no intervention, %
	2021	2022	2023	2024	2025	All		
**Corrections population**								
None^a^	296 (274-305)	261 (239-271)	245 (221-255)	231 (207-241)	226 (202-237)	1259 (1144-1309)	NA	NA
XR-naltrexone only, 66% uptake	271 (253-284)	257 (239-271)	241 (222-255)	227 (209-242)	223 (203-238)	1219 (1128-1288)	40 (16-50)	3.2
All 3 MOUDs, 66% uptake	245 (226-253)	249 (228-258)	237 (215-245)	225 (203-233)	220 (198-230)	1176 (1072- 1216)	83 (72-91)	6.6
**Massachusetts population**								
None^a^	1967 (1901-2294)	2047 (1966-2395)	2136 (2043-2505)	2228 (2129-2617)	2280 (2174-2668)	10 658 (10 200-12 479)	NA	NA
XR-naltrexone only, 66% uptake	1953 (1894-2271)	2030 (1965-2363)	2116 (2045-2470)	2207 (2124-2580)	2257 (2167-2640)	10 563 (10 206-12.316)	95 (85-169)	0.9
All 3 MOUDs, 66% uptake	1940 (1877-2268)	2011 (1935-2357)	2096 (2011-2462)	2185 (2091-2574)	2234 (2134-2623)	10 466 (10 041-12 276)	192 (156-200)	1.8

Abbreviations: MOUD, medications for opioid use disorder; NA, not applicable; UI, uncertainty interval; XR, extended-release.

^a^ Indicates reference category.

The mean discounted lifetime cost per person, from the health care sector perspective, of no MOUD was $302 781 (95% UI, $289 649-$321 891), generating 11.12 (95% UI, 10.68-11.72) discounted QALYs per person (Table 3). Compared with no MOUD, the all-MOUD strategy resulted in an additional mean cost of $852 (95% UI, $14-$1703) per person and an additional gain in QALYs of 0.12 (95% UI, 0.10-0.17) per person, resulting in an ICER of $7252 (95% UI, $140-$10 018) per QALY gained. Compared with the all-MOUD strategy, the XR-naltrexone–only strategy resulted in an additional discounted cost of $2723 (95% UI, $141-$5244) per person but generated 0.04 (95% UI, −0.07 to −0.03) fewer QALYS than the all-MOUD strategy, making it a dominated strategy.

**Table 3.** Costs and QALYs in a Simulated Closed Cohort of Individuals Starting in Corrections in Massachusetts

Intervention	Discounted cost per person, $ (95% UI)	Change in discounted cost per person, $ (95% UI)	Discounted QALY per person (95% UI)	Change in discounted QALY per person (95% UI)	ICER, $ (95% UI)
None^a^	302 781 (289 649 to 321 891)	NA	11.12 (10.68 to 11.72)	NA	NA
All 3 MOUDs, 66% uptake	303 633 (290 268 to 321 273)	852 (14 to 1703)	11.24 (10.80 to 11.82)	0.12 (0.10 to 0.17)	7252 (140-10 018)
XR-naltrexone only, 66% uptake	306 356 (291 143 to 322 100)	2723 (141 to 5244)	11.20 (10.73 to 11.79)	−0.04 (−0.07 to −0.03)	Dominated

Abbreviations: ICER, incremental cost-effectiveness ratio; MOUD, medications for opioid use disorder; NA, not applicable; QALYs, quality-adjusted life-years; UI, uncertainty interval; XR, extended-release.

^a^ Indicates reference category.

### Open Cohort Representing the Massachusetts Population

Among the open cohort simulating the Massachusetts population, total treatment starts were higher at both 1 and 5 years under either intervention strategy (eTable 22 in Supplement 1). By 5 years, there were an additional 28 370 (95% UI, 868-56 033) MOUD treatment initiations, mostly XR-naltrexone, under the strategy of offering XR-naltrexone only during incarceration. By 5 years, there were an additional 32 776 (95% UI, 30 943-34 366) MOUD treatment starts in the all-MOUD strategy, primarily due to increased methadone and buprenorphine initiations.

Overdose deaths were lower at 1 and 5 years under either intervention compared with the no-MOUD scenario (Table 2). Under the no-MOUD scenario, the model estimated 10 658 (95% UI, 10 200-12 479) overdose deaths at 5 years. In the XR-naltrexone–only strategy, a total of 95 (95% UI, 85-169) deaths were averted over the 5-year period from 2021 to 2025, or 0.9% of total overdose deaths in Massachusetts. In the all-MOUD strategy, a total of 192 (95% UI, 156-200) deaths were averted between 2021 and 2025 (a 1.8% decrease compared with no MOUD) (Table 2 and eFigure 5 in Supplement 1).

Overall, the projected 5-year budgetary impact to the Massachusetts health care system of the no MOUD approach from the health care sector perspective was $28.79 (95% UI, $26.9-$30.7) billion, and from a modified societal perspective it was $29.40 (95% UI, $26.61-$31.55) billion (eTable 23 in Supplement 1). The higher cost from the modified societal perspective reflects $607 million cost due to incarceration.

The budgetary impact of the XR-naltrexone–only strategy was $28.96 (95% UI, $26.95-$30.94) billion from the health care sector perspective and $29.56 (95% UI, $27.53-$31.55) billion from the modified societal perspective. The XR-naltrexone–only strategy reduced person-time spent in carceral settings by 1%, corresponding to savings of $6 million dollars in criminal justice costs.

**Figure 2.**
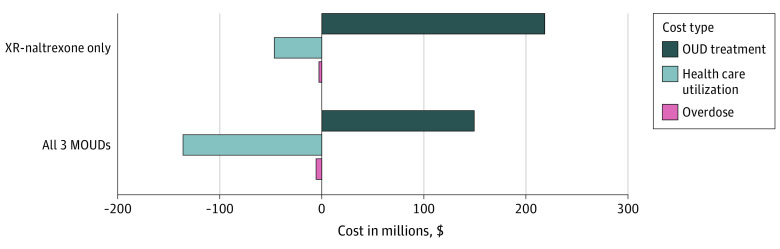
Total Undiscounted Cost Difference of Offering Medications for Opioid Disorder (MOUD) Compared With No Intervention Over 5 Years, 2021 to 2025 Open cohort represents the population of Massachusetts. XR indicates extended release.

The budgetary impact of the all-MOUD strategy was $28.80 (95% UI, $26.00-$30.90) billion, assuming the health care sector perspective, and $29.40 (95% UI, $27.53-$31.32) billion assuming modified societal perspective. The all-MOUD strategy reduced time in carceral setting by 2% compared with no MOUD ($12 million in savings on incarceration) and by 1% compared with XR-naltrexone only ($6 million in savings on incarceration). Thus, while both of the MOUD treatment strategies increased total spending, the nature of that spending shifted toward paying for therapy and away from paying for complications of drug use and overdose (Figure 2).

### Sensitivity Analysis

In deterministic sensitivity analysis, the rate of successful linkage to OUD care after release from incarceration emerged as the most important factor affecting cost-effectiveness conclusions (eTables 24 and 25 in Supplement 1). Still, the all-MOUD strategy remained cost-effective unless the linkage rates fell substantially to 19% for buprenorphine (base case linkage rate, 48%) and 33% for methadone (base case linkage rate, 82%). Ranging the uptake of MOUD had little effect on cost-effectiveness conclusions but did affect the number of overdose deaths prevented. Initiation of XR-naltrexone treatment would have to increase to at least 93% (base-case initiation rate, 66%) for an XR-naltrexone–only strategy to avert more deaths than the all-MOUD strategy (eFigure 6 in Supplement 1). The XR-naltrexone strategy was dominated across all feasible parameter values. Only when we both decreased the cost of XR-naltrexone in corrections by at least 90% and increased the linkage to XR-naltrexone after corrections to 72% (a relative increase of 61% compared with base-case) did the XR-naltrexone–only strategy become undominated. Further, XR-naltrexone was dominated in 72% of probabilistic sensitivity analysis simulations. Cost-effectiveness conclusions were robust to broad sensitivity analysis on all other parameters, including the cost of delivering MOUD in correctional settings (eTable 24 and eFigure 7 in Supplement 1).

## Discussion

In this economic evaluation and modeling study, we found that initiating and continuing MOUD treatment during incarceration could avert a substantial number of opioid overdose deaths at a relatively low cost to the Department of Corrections and Medicaid ($8 million over 5 years) and would be a highly cost-effective intervention. Notably, the XR-naltrexone–only strategy, which is the most commonly used strategy in US correctional systems, was dominated, meaning it both cost more and averted fewer deaths than offering all 3 MOUDs. Despite weaker evidence as to its effiectiveness,^19^ primarily due to low retention, XR-naltrexone is the most widely offered MOUD in correctional settings due to concerns about diversion and views that treatment with agonist medications is substituting one addiction for another.^49^ Still, low retention is a challenge for all 3 medications. Thus, implementing MOUD programs in correctional settings may require education on the efficacy of methadone and buprenorphine and initiatives to combat stigma.

Multiple factors contributed to the XR-naltrexone–only strategy being dominated by the all-MOUD strategy. First, both buprenorphine and methadone have higher linkage to community care post incarceration than does XR-naltrexone, though exactly why is unclear.^7^ Second, among those who do link to community care, retention for both buprenorphine and methadone treatment is better than retention for XR-naltrexone. As a result, the all-MOUD strategy resulted in more than twice the number of people retained in treatment 6 months after release compared with the XR-naltrexone–only strategy. Finally, XR-naltrexone is also the most expensive MOUD, despite the medication’s limitations described previously.

Importantly, while an XR-naltrexone–only strategy was dominated by one offering all 3 MOUDs, for any individual patient, XR-naltrexone may be a reasonable choice. Therefore, consistent with the 2019 National Academies of Sciences, Engineering, and Medicine Consensus Report^13^ and the Massachusetts US Attorney’s 2022 settlement agreement with Massachusetts carceral facilities,^14^ all 3 FDA-approved MOUDs should always be offered.

Our findings are consistent with existing modeling and empirical data. A microsimulation modeling study by Macmadu et al^12^ similarly showed a substantial decrease in overdose deaths with MOUD implementation in correctional facilities in Rhode Island. An empirical study of the first year of an all-MOUD policy in Rhode Island^50^ was also associated with a substantial decrease in opioid overdoses statewide, including a remarkable 61% decrease in postincarceration overdose deaths. Our model estimates for a similar policy in Massachusetts were more modest, however, which may indicate that the actual number of deaths averted may be even higher with clinical implementation than what this model estimates.

In addition, while we did not simulate outcomes stratified by race or ethnicity, we note that improving access by providing MOUD in jails and prisons may be an equity-promoting strategy. Recent data reveal incipient inequities in overdose fatalities by race and ethnicity in Massachusetts,^51^ with increasing overdose death disparities nationally.^52,53^ American Indian or Alaska Native, Black, and Latinx individuals are more likely to be incarcerated in the US^54^; therefore, if equitably applied, a corrections-based MOUD program could increase MOUD access for these populations and begin to mitigate racial and ethnic disparities in overdose mortality.

### Strengths and Limitations

This study has several strengths, including the use of Massachusetts-specific data on individuals with OUD and parameters informed by studies of existing corrections-based MOUD programs. This analysis is also subject to several limitations. First, the factors that affect overdose are complex, and we cannot simulate all such elements. Similarly, some model parameters remain uncertain, or may not be accurate for every context. For example, adherence to buprenorphine even among those receiving prescriptions can vary in a way that affects outcomes, though we did not factor this into the current model.^55^ That being said, while the model does not simulate the details of MOUD adherence, the data we used to populate the model likely did include some individuals who were engaged with MOUD, but not 100% adherent, mitigating the impact of this bias. In addition, this simulation did not include potential benefits and costs of cognitive behavioral therapy (CBT) alongside MOUD provision. While existing evidence does not identify an independent benefit for CBT,^56^ it is generally accepted that best practice includes CBT alongside MOUD provision. Future work could investigate the potential costs and benefits of including CBT as 1 component of MOUD treatment.

While there is remaining uncertainty to decision-making, we performed extensive sensitivity analyses to test the bounds of the estimated impact and quantify the uncertainty. While absolute projections of deaths are uncertain, the relative costs and benefits of strategies are likely robust. Therefore, the qualitative policy conclusions of this analysis—that MOUD in carceral settings would save lives and provide good value for the money invested—are likely robust. In addition, we simulated care delivery in Massachusetts, and overdose and cost projections may not generalize to other states. Still, the incremental comparisons between strategies and qualitative messages of the analysis likely are robust.

## Conclusions

The findings of this economic evaluation and modeling study suggest that offering MOUD during incarceration could prevent opioid overdose deaths, with a strategy including all 3 forms of MOUD being particularly impactful and cost-effective. Importantly, the analysis thereby demonstrates that providing only XR-naltrexone at release, a commonly used strategy, is a relatively poor use of limited resources, due to both retention challenges and XR-naltrexone’s high cost. Given escalating overdose deaths—and the increasing burden of overdose in racial and ethnic minority communities, which are also disproportionately affected by incarceration—correctional facilities should urgently implement comprehensive MOUD programs as part of a suite of state and national policies to combat the opioid epidemic.

## Notes

Supplement 1. **eAppendix.** Model Structure, Costs, Corrections-Specific Data, and Calibration **eFigure 1.** Core Simulation **eFigure 2.** RESPOND’s Care Delivery Module With Project-Specific Strategies Represented **eTable 1.** No-Treatment and Posttreatment: Opioid Use Disorder Transition Parameters **eTable 2.** Opioid Overdoses in MA: Calibration Targets for the Empirical Model **eTable 3.** Opioid Overdose Rates by Age, Sex, Year, and Type of OUD **eTable 4.** Multipliers of Overdose Rates by Treatment **eTable 5.** Probabilities of Fatal Overdose **eTable 6.** Overdose Excluded Weekly Death Probabilities Derived From Life-Tables **eTable 7.** Standardized Mortality Rates (SMRs) **eTable 8.** Weekly Transition Probabilities From No Treatment to Treatment **eTable 9.** Treatment Initiation Effects Parameters: Immediate Transition Probabilities Modeling Movement Between OUD States When Movement Between Treatment States Occurs **eFigure 3.** Core Simulation Within OUD Treatment Episodes (Blocks) **eTable 10.** Transition Probabilities Between Active and Nonactive Opioid Use While Engaged With Treatment **eTable 11.** Weekly Transition Probabilities From Treatment to Posttreatment **eTable 12.** Initializing Cohort Parameters **eTable 13.** Entering Cohort Parameters **eTable 14.** Entering Cohort Counts by Year **eTable 15.** Cost of Naltrexone Treatment for Opioid Use Disorder **eTable 16.** Cost of Buprenorphine Treatment for Opioid Use Disorder **eTable 17.** Cost of Methadone Treatment for Opioid Use Disorder **eTable 18.** Cost of Nonfatal Overdose **eTable 19.** Cost of Fatal Overdose **eTable 20.** 1- and 5-Year MOUD Initiations (Closed Cohort) **eTable 21.** Percent Retained on MOUD at 6 Months Post Release (Closed Cohort) **eTable 22.** 1- and 5-Year Treatment Starts (MA Population Open Cohort) **eTable 23.** Undiscounted Budgetary Impact for MA Over 5 Years of Intervention (2021-2025), Total Cost in Millions of USD **eTable 24.** Sensitivity Analysis Using Multiplicative Approach: ICER Table **eTable 25.** Deterministic Sensitivity Analyses **eFigure 4.** Overdose Deaths Among MA Population During 5-Year Intervention with Varying Initiation of XR-NTX Strategy **eFigure 5.** Fatal Overdoses Averted, Compared With No Intervention **eFigure 6.** Cost-effectiveness Acceptability Curve (CEAC) of Tested Strategies **eFigure 7.** Fatal Overdoses as a Model Calibration Target
eReferences

Supplement 2.
Data Sharing Statement
