# Supplementary material for: Estimated Costs and Outcomes Associated With Use and Nonuse of Medications for Opioid Use Disorder During Incarceration and at Release in Massachusetts
Source: JAMA Netw Open. 2023 Apr 14;6(4):e237036. doi: 10.1001/jamanetworkopen.2023.7036 (PMC10105308; doi:10.1001/jamanetworkopen.2023.7036)
Supplement: Supplement 1. — eAppendix. Model Structure, Costs, Corrections-Specific Data, and Calibration eFigure 1. Core Simulation eFigure 2. RESPOND’s Care Delivery Module With Project-Specific Strategies Represented eTable 1. No-Treatment and Posttreatment: Opioid Use Disorder Transition Parameters eTable 2. Opioid Overdoses in MA: Calibration Targets for the Empirical Model eTable 3. Opioid Overdose Rates by Age, Sex, Year, and Type of OUD eTable 4. Multipliers of Overdose Rates by Treatment eTable 5. Probabilities of Fatal Overdose eTable 6. Overdose Excluded Weekly Death Probabilities Derived From Life-Tables eTable 7. Standardized Mortality Rates (SMRs) eTable 8. Weekly Transition Probabilities From No Treatment to Treatment eTable 9. Treatment Initiation Effects Parameters: Immediate Transition Probabilities Modeling Movement Between OUD States When Movement Between Treatment States Occurs eFigure 3. Core Simulation Within OUD Treatment Episodes (Blocks) eTable 10. Transition Probabilities Between Active and Nonactive Opioid Use While Engaged With Treatment eTable 11. Weekly Transition Probabilities From Treatment to Posttreatment eTable 12. Initializing Cohort Parameters eTable 13. Entering Cohort Parameters eTable 14. Entering Cohort Counts by Year eTable 15. Cost of Naltrexone Treatment for Opioid Use Disorder eTable 16. Cost of Buprenorphine Treatment for Opioid Use Disorder eTable 17. Cost of Methadone Treatment for Opioid Use Disorder eTable 18. Cost of Nonfatal Overdose eTable 19. Cost of Fatal Overdose eTable 20. 1- and 5-Year MOUD Initiations (Closed Cohort) eTable 21. Percent Retained on MOUD at 6 Months Post Release (Closed Cohort) eTable 22. 1- and 5-Year Treatment Starts (MA Population Open Cohort) eTable 23. Undiscounted Budgetary Impact for MA Over 5 Years of Intervention (2021-2025), Total Cost in Millions of USD eTable 24. Sensitivity Analysis Using Multiplicative Approach: ICER Table eTable 25. Deterministic Sensitivity Analyses eFigure 4. Overdose Deaths Among MA Po [file jamanetwopen-e237036-s001.pdf]

Supplementary Online Content

Chatterjee A, Weitz M, Savinkina A, et al. Estimated costs and outcomes associated with use and nonuse of medications for opioid use disorder during incarceration and at release in Massachusetts. *JAMA Netw Open*. 2023;6(4):e237036. doi:10.1001/jamanetworkopen.2023.7036

eAppendix. Model Structure, Costs, Corrections-Specific Data, and Calibration

eFigure 1. Core Simulation

eFigure 2. RESPOND's Care Delivery Module With Project-Specific Strategies Represented

eTable 1. No-Treatment and Posttreatment: Opioid Use Disorder Transition Parameters

eTable 2. Opioid Overdoses in MA: Calibration Targets for the Empirical Model

eTable 3. Opioid Overdose Rates by Age, Sex, Year, and Type of OUD

eTable 4. Multipliers of Overdose Rates by Treatment

eTable 5. Probabilities of Fatal Overdose

eTable 6. Overdose Excluded Weekly Death Probabilities Derived From Life-Tables

eTable 7. Standardized Mortality Rates (SMRs)

eTable 8. Weekly Transition Probabilities From No Treatment to Treatment

eTable 9. Treatment Initiation Effects Parameters: Immediate Transition Probabilities Modeling Movement Between OUD States When Movement Between Treatment States Occurs

eFigure 3. Core Simulation Within OUD Treatment Episodes (Blocks)

eTable 10. Transition Probabilities Between Active and Nonactive Opioid Use While Engaged With Treatment

eTable 11. Weekly Transition Probabilities From Treatment to Posttreatment

eTable 12. Initializing Cohort Parameters

eTable 13. Entering Cohort Parameters

eTable 14. Entering Cohort Counts by Year

eTable 15. Cost of Naltrexone Treatment for Opioid Use Disorder

eTable 16. Cost of Buprenorphine Treatment for Opioid Use Disorder

eTable 17. Cost of Methadone Treatment for Opioid Use Disorder

eTable 18. Cost of Nonfatal Overdose

eTable 19. Cost of Fatal Overdose

eTable 20. 1- and 5-Year MOUD Initiations (Closed Cohort)

© 2023 Chatterjee A et al. *JAMA Network Open*.

eTable 21. Percent Retained on MOUD at 6 Months Post Release (Closed Cohort)

eTable 22. 1- and 5-Year Treatment Starts (MA Population Open Cohort)

eTable 23. Undiscounted Budgetary Impact for MA Over 5 Years of Intervention (2021-2025), Total Cost in Millions of USD

eTable 24. Sensitivity Analysis Using Multiplicative Approach: ICER Table

eTable 25. Deterministic Sensitivity Analyses

eFigure 4. Overdose Deaths Among MA Population During 5-Year Intervention with Varying Initiation of XR-NTX Strategy

eFigure 5. Fatal Overdoses Averted, Compared With No Intervention

eFigure 6. Cost-effectiveness Acceptability Curve (CEAC) of Tested Strategies

eFigure 7. Fatal Overdoses as a Model Calibration Target

eReferences

This supplementary material has been provided by the authors to give readers additional information about their work.

© 2023 Chatterjee A et al. *JAMA Network Open*.

eAppendix. Model Structure, Costs, Corrections-Specific Data, and Calibration

A. INTRODUCTION

The growing prevalence of Opioid Use Disorder (OUD) has resulted in an increase in opioid overdoses in the United States. Overdose is the leading cause of premature death among Americans under the age of 50 and has increased by more than 2.5 times between 1999 and 2015. Although evidence-based treatments are available for treating OUD, these treatments are under-utilized, thus the impact of opioids on the United States' population persists.

Researchers and policy makers have made efforts to create feasible action plans for reducing the prevalence of OUD. Unfortunately, most policy makers do not have the evidence needed for informing and implementing system-level change. System-level thinking investigates how systems operate and how they can be modified to produce desired outcomes. At this time, data on system-level interventions for OUD are limited and inconsistent.

In an effort to fill the knowledge gap, simulation modeling can be used to integrate data from multiple sources to translate outcomes from clinical studies to policy-relevant data about population health and cost. By simulating state-level behaviors and practices related to OUD, we can project and evaluate the impact of relevant interventions and policies on public health outcomes and costs, hence informing practice and policy decisions to combat OUD.

The **R**esearching **E**ffective **S**trategies to **P**revent **O**pioid **D**eath (RESPOND) model is a state-transition, cohort-based model that simulates populations with high-risk opioid use in a state, including the natural history of opioid use disorder, movement on and from opioid treatment, and overdose. The model provides outputs and projections that decision-makers can use to evaluate and modify care delivery systems to match their local epidemics and available resources.

Model inputs and parameters are adaptable to users' needs, namely, to represent heterogeneous populations, different dynamics of the drug overdose epidemic, and the effectiveness of intervention strategies in the prevention of opioid-related harms. The user, for example, can customize among other things, the demographics, time in each cycle, transition probabilities between health statuses and treatment states, and the number of health states included in the model in order to represent different structure and disease dynamics of the underlying population.

© 2023 Chatterjee A et al. *JAMA Network Open*.

B. MODEL STRUCTURE

B.1 Overview

RESPOND is a state-transition, cohort-based model that simulates the population living within a jurisdiction and who have high-risk opioid use [1,2]. Typically, RESPOND simulates the population of a state, but it can also simulate a smaller area, such as a town or rural community, depending on the model parameter values. The model employs a Markov process with a weekly cycle length to accurately reflect population dynamics, clinical progression, and treatment of opioid use disorder.

The model structure comprises four main components: 1) population dynamics, 2) natural history of OUD, 3) care delivery, and 4) mortality.

The population dynamics modules simulate the epidemiology and demography of the opioid epidemic. The user can create either an open or a closed cohort simulation. In an open cohort simulation, new population “arrives” to the simulation in every time step such that the total population in the model always reflects the size of the total population with OUD living in that jurisdiction. The arrival rate represents both the development of new opioid use disorder and migration into the state among those with existing opioid use. In a closed cohort, no cohort members enter the simulation and the size of the population in the simulation dwindles over time as cohort members die.

The core simulation (***Supplemental Figure 1***) of the RESPOND model involves the simulation of the natural history of opioid use disorder as a relapsing and remitting disease over a lifetime. RESPOND simulates OUD as a series of transitions between four health states of opioid use: 1) active, non-injection, 2) non-active, non-injection, 3) active injection, and 4) non-active, injection opioid use. In each time-step of the simulation, population fractions move between opioid use states. The definitions of “active” and “injection” opioid use can vary (but must be pre-specified) depending on the users’ needs and available information. In the RESPOND Massachusetts base case, “active” opioid use is defined as any reported use in the previous seven days. “Injection” opioid use reflects any injection in the preceding seven days (a person who is both injecting and using oral opioids would be categorized as “injection” in RESPOND).

The care delivery module (***Supplemental Figure 2)*** of RESPOND simulates OUD treatment and includes four treatment types: 1) outpatient buprenorphine (Bup), 2) outpatient injectable naltrexone (Ntx), 3) outpatient methadone (Mmt) maintenance, and 4) inpatient acute drug detoxification (detox). The model is adaptable to additional intervention types to better reflect local conditions and evolutions in the

© 2023 Chatterjee A et al. *JAMA Network Open*.

treatment field. For this study, a fifth block was added to capture the state of Corrections. In general, treatment episodes tend to decrease movement into active drug use, increase movement into non-active drug use, and have an independent effect on overdose rates conditional on active drug use. When population disengages from a treatment and is lost to follow-up, those people enter a corresponding "post-treatment state". The post-treatment state is a fixed interval during which relapse to active drug use is high, tolerance to opioids is lower than before treatment, and the risk of drug overdose among those actively using opioids is higher than it is in the no treatment state. The post-treatment state represents the period of vulnerability and excess overdose observed in real-world settings among patients who have recently relapsed to opioid use after a period of sustained abstinence.

The mortality module simulates both drug-related and competing risks deaths. RESPOND simulates overdose mortality by first simulating overdose incidence as a function of age and type of drug use (injection vs. non-injection use). Next, the model simulates a probability of death conditional on having had an opioid overdose. The model simulates competing causes of death through the use of standardized mortality ratios that are a function of age, sex, and type of opioid use (injection vs. non-injection).

The primary model outputs are: 1) All-cause mortality, 2) Overdose mortality, and 3) Number of people on treatment.

The simulation process is as follows: At simulation start, the model initiates a cohort of people currently living with OUD in the jurisdiction of interest. Based on data from that jurisdiction, the model assigns the current population to a drug use state, as well as a treatment block, such that the simulated population, including the prevalence of OUD treatment, reflects the status quo. Moving forward through simulated time, the sequence of simulation steps are: 1) aging of the population, 2) arrival of new population, 3) transition between OUD drug use states, 4) transitions into and out of treatment, 5) overdose, and 6) death. At the end of this sequence of processes, the model advances simulated time by one cycle (week) and repeats the process. The simulation continues until a time horizon assigned by the user.

Full model schematics are available at https://www.syndemicslab.org/respond.

Following sections of this document provide details of model parameters for a specific use case of the model that simulates the OUD population of the state of Massachusetts from the end of year 2012-2015 with an open cohort.

© 2023 Chatterjee A et al. *JAMA Network Open*.

eFigure 1. Core Simulation

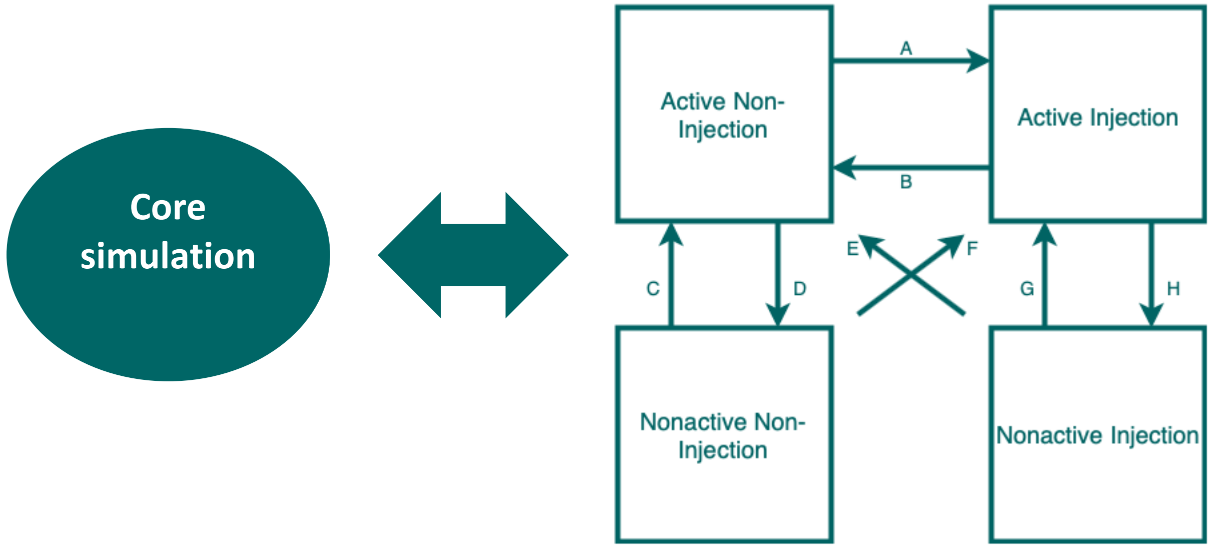

© 2023 Chatterjee A et al. *JAMA Network Open*.

eFigure 2. RESPOND's Care Delivery Module With Project-Specific Strategies Represented

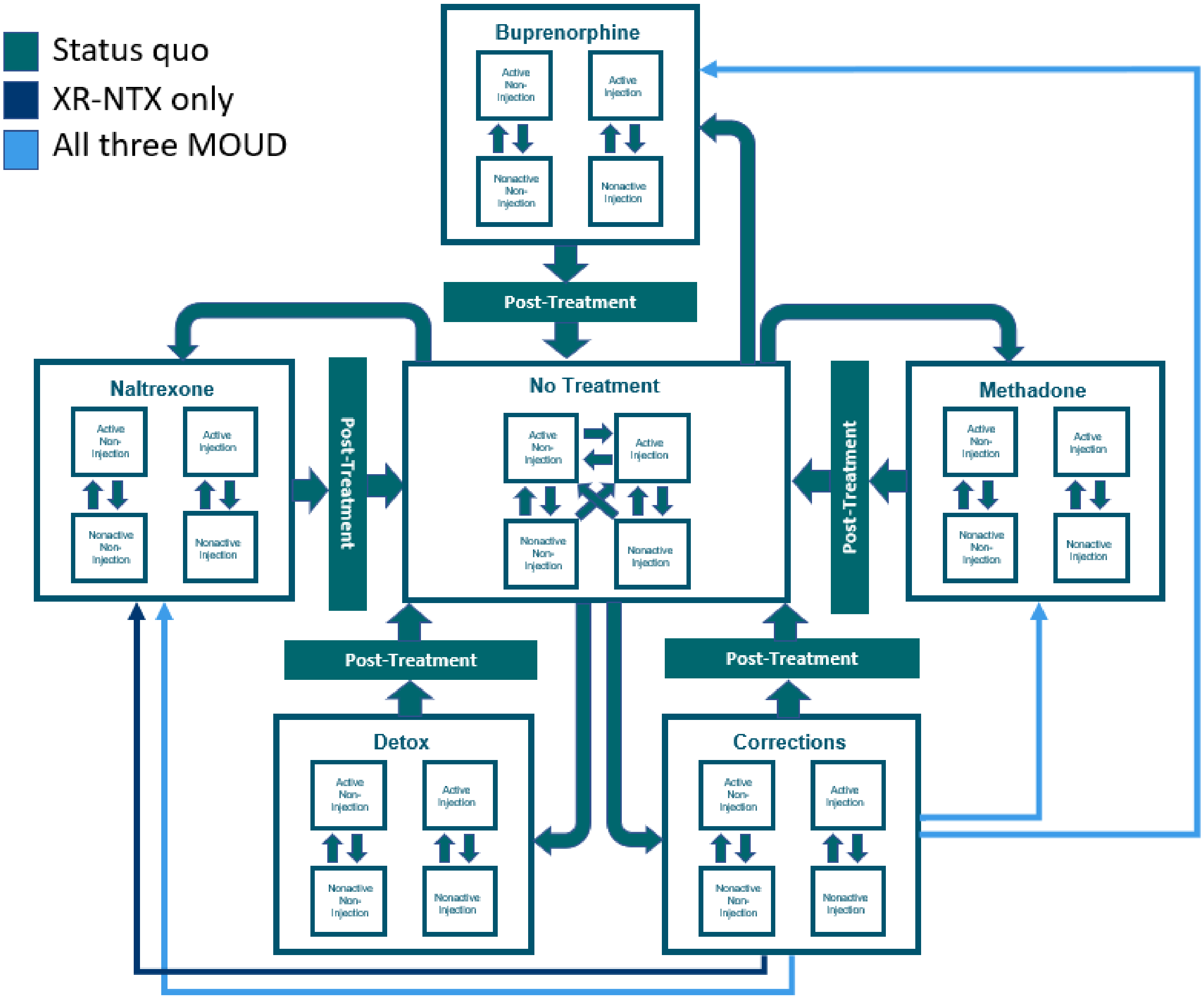

B.1.1 Natural History of OUD

RESPOND simulates opioid use as a series of transitions through four opioid use health states: 1) Non-Active and 2) Active non-injection use, as well as 3) Non-Active and 4) Active injection use ([*Figure 1*]()). Throughout the simulation, there is a multi-directional movement between OUD states.

© 2023 Chatterjee A et al. *JAMA Network Open*.

Transitions between drug use compartments impact four important outcomes: 1) risk of overdose, 2) risk of death from competing causes, 3) health care utilization (cost), and 4) quality of life.

The primary sources of data for substance use transitions are studies from the medical literature.

Structural Assumptions:

- OUD is a remitting and relapsing process over a lifetime. There is no health state of OUD cure or permanent recovery.
- Transitions between OUD health states are not time updated.

Methodological Notes:

Supplemental Table 1 presents the key parameters related to OUD transitions for no treatment.

- All Confidence Intervals (CIs) are 95%, namely calculated at α=5% level of significance.
- CIs for proportions p_E, p_F, p_B, p_H, and p_D are calculated using the normal approximation to binomial proportions.
- CIs for rates R_A, R_C, and R_D are provided from the manuscript and calculated assuming Poisson distribution.
- Weekly rates and proportions, calculated from the respective overall estimates, are converted to weekly transition probabilities as indicated in the "Method" column in **Supplemental Table 1**.

eTable 1. No-Treatment and Posttreatment: Opioid Use Disorder Transition Parameters

Parameter	Description	Value	Method	Source
**No Treatment (ρ_N)**				
R_A	Rate of active non-injection to active injection	4.6 per 100 PY (3.0, 6.6)	Direct from reference	Neaigus, A., et al. [11]
**P_A**	**Probability of active non-injection to active injection**	**0.000884 (0.000577, 0.001268)**	**Calculated from R_A: P_A= 1-exp{R_A/52}**	
R_C	Rate of non-active non-injection to active non-injection	16 per 100 PY (12.0, 20.5)	Direct from reference	
R_G	Rate of non-active injection to active injection			
**P_C**	**Probability of non-active non-injection to active non-injection**	**0.00307**		

© 2023 Chatterjee A et al. *JAMA Network Open*.

**P_G**	**Probability of non-active injection to active injection**	**(0.00230, 0.00393)**	**Calculated from Rate(R_C): P= 1-exp{R_C/52}**	
p_B	Proportion of active injection to active non-injection	0.34		
**P_B**	**Probability of active injection to active non-injection**	**0.00067 (0.00054 , 0.0008)**	**Calculated from p_B: 1-exp(x) where x = ln(1-p_B)/(12*52)**	
p_E	Proportion of non-active injection to active non-injection	0.13	Direct from reference	Shah, N.G., et al. [12]
p_F	Proportion of non-active non-injection to active injection			
**P_E**	**Probability of non-active injection to active non-injection**	**0.000223 (0.000115 , 0.00034)**	**Calculated from p_E: 1-exp(x) where x = ln(1-p_E)/(12*52)**	
**P_F**	**Probability of non-active non-injection to active injection**			
p_D	Proportion of active non-injection to non-active non-injection	0.03 (0.0175 , 0.0425)	Direct from reference	Nosyk, B., et al. [13]
p_H	Proportion of active injection to non-active injection			
**P_D**	**Probability of active non-injection to non-active non-injection**	**0.00058 (0.00032 , 0.00085)**	**Calculated from p_D: 1-exp(x) where x = ln(1-p_D)/(52)**	
**P_H**	**Probability of active injection to non-active injection**			
**Post-Treatment (ρ_P)**				
P_A, P_B, P_D, P_E, P_F, P_H,	Same estimates with no-treatment.			
p^*_C	Proportion of non-active non-injection to active non-injection	0.65	Direct from reference	Bailey et al. [14]
p^*_G	Proportion of non-active injection to active injection			
**P_C**	**Probability of non-active non-injection to active non-injection**	**0.2308**	**Calculated from p: 1-exp(x) where x = ln(1-p)/4**	
**P_G**	**Probability of non-active injection to active injection**			

- p^*_C and p^*_G indicate the percentage of people relapsed within a month of discharge (after inpatient detoxification).
- The denominators for calculating weekly probabilities depend on whether the respective available proportion or rate estimates are yearly or monthly.

B.1.2 Overdose

Every person who is actively using opioids faces the risk of overdose. The probability of overdose depends on age, sex, and route of drug use (injection vs. non-injection). The simulation has no memory of

© 2023 Chatterjee A et al. *JAMA Network Open*.

past overdose events and does not include an elevated risk of repeat overdose after experiencing a first overdose event.

Structural Assumptions:

- Experiencing overdose has no independent impact on current or future opioid use behaviors.
- Only the population that is in an active opioid use state faces the risk of overdose.
- The risk of overdose is different between no-treatment, treatment, and post-treatment episodes.
- The risk of overdose is lower while engaged in treatment compared to not-engaged, even among the population who are actively using drugs while engaged with treatment.

Methodological Notes:

Counts of overdose are a target for model calibration. ***Supplemental Table 2*** provides the empirically observed overdose fatalities from MA PHD.

eTable 2. Opioid Overdoses in MA: Calibration Targets for the Empirical Model

Year	Age	Sex	Total number of people with opioid overdose	Number of fatal opioid overdose	Number of people with non-fatal overdose	Total overdoses
2013	10-19	Male	88	7	86	89
2013	10-19	Female	77	1	76	77
2013	20-24	Male	542	50	507	547
2013	20-24	Female	346	18	337	350
2013	25-39	Male	1929	264	1746	1966
2013	25-39	Female	975	99	899	995
2013	40-54	Male	1147	240	966	1178
2013	40-54	Female	776	121	673	791
2013	55+	Male	705	70	647	713
2013	55+	Female	630	30	596	631
2014	10-19	Male	112	13	107	113
2014	10-19	Female	101	1	101	101
2014	20-24	Male	823	75	778	833
2014	20-24	Female	502	33	482	508
2014	25-39	Male	3174	421	2898	3268
2014	25-39	Female	1415	141	1313	1443

© 2023 Chatterjee A et al. *JAMA Network Open*.

2014	40-54	Male	1607	314	1368	1643
2014	40-54	Female	941	143	816	961
2014	55+	Male	913	105	825	928
2014	55+	Female	754	48	704	756
2015	10-19	Male	114	16	109	116
2015	10-19	Female	95	3	93	96
2015	20-24	Male	800	97	742	827
2015	20-24	Female	465	37	446	478
2015	25-39	Male	3804	545	3411	3914
2015	25-39	Female	1650	157	1537	1686
2015	40-54	Male	1863	357	1594	1919
2015	40-54	Female	973	142	871	1002
2015	55+	Male	1025	150	913	1045
2015	55+	Female	821	58	774	828

B.1.2.1 Overall (non-block specific) overdose rates

Yearly rate $R_{OD,t}$ of overdose at time t for people not engaged in treatment is calculated as:

$$R_{OD,t} = \frac{N_{OD,t}}{N_{OUD,t} + \frac{1}{2} \cdot N_{enter,t}} \times 1\ PY \qquad (4)$$

for years t= 2013, 2014, 2015, assuming that each person contributes 1 person/year where:

$N_{OD,t}$: number overdose cases at time t

$N_{OUD,t}$: OUD cohort size at time t

$N_{enter,t}$: entering cohort size at time t

Therefore, weekly overall overdose rate from anywhere in the model $o(t) = {}^{R_{OD,t}}/_{52}$.

Supplemental Table 3 presents point estimates of the overdose rates by age group, sex, OUD type, and year.

eTable 3. Opioid Overdose Rates by Age, Sex, Year, and Type of OUD

**Age**	**Sex**	**OUD**	**Years***		
			**2013**	**2014**	**2015**
10-19	Male	Active_Noninjection	0.000112076	0.000409791	0.000141081
10-19	Male	Active_Injection	0.000674857	0.002467526	0.000849508
10-19	Female	Active_Noninjection	0.000162217	0.000458584	0.000417609
10-19	Female	Active_Injection	0.000976778	0.002761329	0.002514598
20-24	Male	Active_Noninjection	0.001380621	0.000974182	0.000804356
20-24	Male	Active_Injection	0.008313304	0.005865957	0.004843369

© 2023 Chatterjee A et al. *JAMA Network Open*.

20-24	Female	Active_Noninjection	0.001557061	0.001189503	0.000777667
20-24	Female	Active_Injection	0.00937572	0.007162497	0.004682664
25-39	Male	Active_Noninjection	0.000468337	0.000755758	0.00072912
25-39	Male	Active_Injection	0.002820052	0.004550736	0.004390337
25-39	Female	Active_Noninjection	0.00037227	0.000421042	0.000529921
25-39	Female	Active_Injection	0.002241592	0.002535274	0.003190876
40-54	Male	Active_Noninjection	0.000310914	0.000406988	0.000629183
40-54	Male	Active_Injection	0.001872141	0.002450645	0.003788574
40-54	Female	Active_Noninjection	0.000304432	0.000306566	0.000557584
40-54	Female	Active_Injection	0.001833113	0.001845965	0.003357447
55-99	Male	Active_Noninjection	0.000192418	0.000282608	0.000389897
55-99	Male	Active_Injection	0.001158631	0.0017017	0.002347731
55-99	Female	Active_Noninjection	0.00020927	0.000261909	0.000126691
55-99	Female	Active_Injection	0.0012601	0.001577063	0.000762859
* Overdose rates presented are weekly overall fixed rates o(t) for each year.					

Note that RESPOND model simulations has weekly time cycles. Let $\nu_B(\mathrm{t})$ be the overdose rate in a specific block where $B = N, T$ or P for blocks no-treatment, treatment, and post-treatment. Then, weekly overdose probabilities $P_{OD,B,t}$ are calculated from the respective overdose rates as:

$$P_{OD,B,t} = 1 - e^{-\nu_B(\mathrm{t})} \qquad (5)$$

where $B = N, T$ or P.

B.1.2.2 No Treatment

Weekly overdose rates in no treatment $\nu_N(\mathrm{t})$ were calculated by applying a multiplier $\mathbf{m_N} \in \mathbb{R}^+$ on overall overdose rates $\mathrm{o(t)}$ as $\nu_N(\mathrm{t}) = \mathrm{o(t)} \times \mathbf{m_N}$. There were no data available to inform the rate multiplier $\mathbf{m_N}$. Therefore, we decided to calibrate overdose rate multiplier of no treatment block.

B.1.2.3 Overdose While on Treatment

The risk of overdose for people engaged in treatment, is derived by applying a multiplier parameter $\mathbf{m_T}$ to the respective no-treatment $\nu_N(t)$ estimates. i.e., the weekly overdose rate at time t for treatment T is:

$$\nu_T(\mathrm{t}) = \nu_N(t) \times \mathbf{m_T} \qquad (6)$$

where $\nu_N(\mathrm{t})$ is the no-treatment overdose rate at time t, and $\mathbf{m_T} \in (0, 1)$.

© 2023 Chatterjee A et al. *JAMA Network Open*.

eTable 41. Multipliers of Overdose Rates by Treatment

**Treatment** T*	$\mathbf{m_T}$	**95% CI**	**Source**
Buprenorphine	0.405	[0.35 , 0.46]	Morgan et al. [16]
Naltrexone	0.864	[0.42, 1.31] injectable	
Methadone	0.752**	Non-parametric uncertainty distribution from bootstrapping of data	Morgan et al. [16] Sordo et al. [18]

* We assume no overdoses occur in the detox block. Therefore, overdose rate is zero.

** $\mathbf{m_{Meth}} = \mathbf{m_{Bup}} \times \frac{\textit{Meth rate}}{\textit{Bup rate}} = 0.405 \times \frac{2.6}{1.4}$

B.1.2.4 Overdose During the Post-Treatment Period

During the post-treatment period, individuals face a risk of overdose higher than that of people who never initiated a treatment. Therefore, we model post-treatment overdose rates $\nu_P(\mathrm{t})$ with a multiplier $\mathbf{m_P}$ (greater than 1) applied on no-treatment overdose rates such that $\nu_P(\mathrm{t}) = \nu_N(\mathrm{t}) \times \mathbf{m_P}$. There were no data available to inform post-treatment overdose rate multiplier $\mathbf{m_P}$. Therefore, we decided to calibrate overdose rate multiplier of post-treatments.

B.1.3 Mortality

RESPOND simulates mortality through two independent mechanisms, fatal opioid overdose and non-overdose death.

B.1.3.1 Fatal Overdose

The population that experiences overdose then faces a probability of death conditional on having had an overdose. This conditional probability of death, given an opioid overdose, is generalizable to all overdose cases and is therefore not stratified by age, sex, or OUD status. The population that survives an overdose does not change substance use as a result of the overdose. The probability of death conditional on having experienced an overdose is a time updated variable, reflecting changes to drug supply over time. Adjusting the conditional probability of overdose death provides a mechanism to reflect the growing penetration of fentanyl in local drug supplies, which is a major dynamic underlying mounting overdose deaths in the U.S.

© 2023 Chatterjee A et al. *JAMA Network Open*.

The probability $\mathbf{f(t)}$ of fatal overdose at year t is calculated as:

$$\mathbf{f(t)} = \frac{N_{FOD,t}}{N_{OD,t}} \qquad (7)$$

where $N_{FOD,t}$ is the total number of fatal overdoses, and $N_{OD,t}$ is the total number of all-type overdoses.

eTable 5. Probabilities of Fatal Overdose

Year t	$\mathbf{f(t)}$
2013	0.1248 (0.1161, 0.1338)*
2014	0.1251 (0.1179, 0.1324)*
2015	0.1346 (0.1275, 0.1417)*

* Empiricaly calculated 95% CI using the Poisson distribution assumption for both counts in numerator and denominator of Equation 7

B.1.3.2 Competing risks of death (non-overdose mortality)

Competing risks mortality includes deaths from conditions such as infectious endocarditis and sepsis, as well as medical comorbidities that accrue over a lifetime. The general approach to estimating competing risks of death is to apply standardized mortality ratios (SMRs) reflecting elevated mortality among drug users to age-sex stratified actuarial lifetables for the U.S. [19].

eTable 6 presents weekly death probabilities P_{NOD} calculated from lifetables in National Vital Statistic System after excluding overdoses [19].

eTable 6. Overdose Excluded Weekly Death Probabilities Derived From Life-Tables

Age	Sex	Probability of Death
10-14	Male	3.23e-06
10-14	Female	2.33e-06
15-19	Male	1.22e-05
15-19	Female	5.32e-06
20-24	Male	2.20e-05
20-24	Female	7.85e-06
25-29	Male	2.43e-05
25-29	Female	1.00e-05
30-34	Male	2.77e-05
30-34	Female	1.41e-05

© 2023 Chatterjee A et al. *JAMA Network Open*.

35-39	Male	3.37e-05
35-39	Female	1.90e-05
40-44	Male	4.50e-05
40-44	Female	2.83e-05
45-49	Male	6.83e-05
45-49	Female	4.41e-05
50-54	Male	0.000113101
50-54	Female	7.06e-05
55-59	Male	0.000173492
55-59	Female	0.000105002
60-64	Male	0.000253182
60-64	Female	0.000149513
65-69	Male	0.000350328
65-69	Female	0.000223448
70-74	Male	0.000535512
70-74	Female	0.000363913
75-79	Male	0.00084698
75-79	Female	0.000601651
80-84	Male	0.001427294
80-84	Female	0.001055789
85-89	Male	0.002907422
85-89	Female	0.002393584
90-94	Male	0.002907422
90-94	Female	0.002393584
95-99	Male	0.002907422
95-99	Female	0.002393584

Weekly non-overdose death rates R_{NOD} are calculated as

$$R_{NOD} = \ln(1 - P_{NOD}) \times SMR \qquad (1)$$

where SMR is calculated as:

$$\text{SMR} = \frac{\text{N}_{\text{Non-FOD}}}{\text{R}_\text{D} \times \text{N}_{\text{OUD}}} \qquad (9)$$

where $N_{Non\text{-}FOD}$ is the number of observed deaths not due to opioid overdose, R_D is the census death rate, and N_{OUD} is the size of the OUD population based on chapter 55 estimation.

We construct CIs around the SMRs estimates assuming that the $N_{Non\text{-}FOD}$ follows a Poisson distribution and using the normal approximation (***Supplemental Table 7***).

© 2023 Chatterjee A et al. *JAMA Network Open*.

**eTable 7.** Standardized Mortality Rates (SMRs)		
**sex**	**OUD type**	**SMRs (95% CIs)**
Male	Active - Non-injection	1.79(1.58 , 2.00)
Male	Active - Injection	4.41(3.84 , 4.98)
Male	Non-active – Non-injection	1.83(1.15 , 2.50)
Male	Non-active - Injection	4.59(2.71 , 6.46)
Female	Active – Non-injection	2.31(1.99 , 2.63)
Female	Active - Injection	5.67(4.81 , 6.53)
Female	Non-active – Non-injection	2.30(1.29 , 3.31)
Female	Non-active - Injection	5.62(2.87 , 8.37)

Finally, we convert weekly non-overdose death rates to probabilities as $1 - e^{-R_{NOD}}$.

B.1.3.3 Summary of the combined impact of medications for opioid use disorder on all-cause mortality in RESPOND

Medications for opioid use disorder (MOUD) have two independent effects on mortality that combine to provide synergies in the simulation:

1. The population that is engaged with MOUD treatment experiences a net movement toward non-active drug use. Because there is no risk of overdose while not using drugs, MOUD tend to decrease the rate of overdose in the population. In addition, movement out of active drug use states reduces exposure to the high standardized mortality ratios (SMRs) of active drug use and thereby reduce non-overdose mortality as well.
2. Among those who are actively using drugs when taking an MOUD, the MOUD has an independent effect on overdose risk, such that even those who are using have lower risk of death than those who are using drugs while not engaged with MOUD treatment.

B.1.4 Care Delivery

RESPOND models OUD while engaged with treatment using the same 4-state opioid use simulation that it uses to model OUD without treatment. The 4-state OUD simulation is embedded within all treatment episodes (blocks), such that individuals may both remain engaged with treatment, but also experience periods of drug use relapse. Each treatment type has its own bi-directional transition probabilities between active and non-active use. The net movement between active and non-active use while engaged with treatment favors movement to non-active use over time.

© 2023 Chatterjee A et al. *JAMA Network Open*.

RESPOND simulates treatment using the following parameters:

1. Probability of movement onto treatment from no treatment
2. Treatment initiation effect – the probability of ceasing active opioid use immediately after initiating treatment
3. Bi-directional movements between active and non-active opioid use while engaged with treatment
4. Probability of loss to follow-up

The population that is lost to follow-up (disengages from care) must pass through a "post-treatment period" before rejoining the simulation of OUD. The post-treatment period is a four-week time, immediately following discontinuation of a treatment, during which the risk of relapse to drug use is high, as is the risk of overdose. Population that survives the post-treatment period transitions back to the simulation of OUD without treatment.

B.1.4.1 Movement From No-Treatment to Treatment Episodes

Structural Assumptions:

- Only population in active opioid use states seeks OUD treatment. Population that is not currently using opioids does not seek treatment.

The main source of data to inform the probability of transition from no treatment to a treatment episode is the MA PHD.

Methodological Notes:

Let λ_{NT} denotes the weekly transition rates from no-treatment to treatment. Then, weekly transition probability from no-treatment to treatment is calculated from λ_{NT} as:

$$\widehat{\mathbf{P}}_{\mathbf{NoTrt} \to \mathbf{Trt.T}} = 1\text{- exp } \{-\lambda_{NT}\} \qquad (2)$$

Where $\lambda_{NT} = \frac{\widehat{\mathbf{N}}_{\mathbf{Obs,NoTrt} \to \mathbf{Trt.T}}}{\widehat{\mathbf{N}}_{\mathbf{Total,NoTrt} \to \mathbf{Trt.T}}} \times \frac{1}{4}$.

Here $\widehat{\mathrm{N}}_{\mathrm{Obs,NoTrt} \to \mathrm{Trt.T}}$: the observed number of people with OUD who transitioned from no-treatment to treatment T in January 2013

© 2023 Chatterjee A et al. *JAMA Network Open*.

$\widehat{N}_{Total,NoTrt \rightarrow Trt.T}$: the total number of people with OUD "*at risk*" of transitioning from no-treatment to treatment T in January 2013

The weekly transition probability $\widehat{P}_{NoTrt \rightarrow Trt.T}$ is estimated using data from the MA PHD repository[1], and is stratified by age (16 groups: 5-year age-groups from 10-85, and >85years old), sex, and treatment (T= Detox, Mmt, Ntx, and Bup) (***Supplemental Table 8***).

eTable 8. Weekly Transition Probabilities From No Treatment to Treatment

Age	Sex	Transition to Treatment			
		**Detox***	**Methadone**	**Naltrexone**	**Buprenorphine**
		$\widehat{P}_{NoTrt \rightarrow Trt.T} = 1 - e^{-\widehat{\lambda}_{ND}}$	$\widehat{P}_{NoTrt \rightarrow Trt.T} = 1 - e^{-\lambda_{NM}}$	$\widehat{P}_{NoTrt \rightarrow Trt.T} = 1 - e^{-\lambda_{NX}}$	$\widehat{P}_{NoTrt \rightarrow Trt.T} = 1 - e^{-\lambda_{NB}}$
10-14	Male	0.0037	0.0037	0.0037	0.0037
10-14	Female	0.0064	0.0064	0.0064	0.0064
15-19	Male	0.0027	0.0004	0.0016	0.0023
15-19	Female	0.0027	0.0005	0.0016	0.0027
20-24	Male	0.0054	0.0009	0.0012	0.0051
20-24	Female	0.005	0.0016	0.001	0.0057
25-29	Male	0.0056	0.0016	0.0007	0.0062
25-29	Female	0.0042	0.0028	0.0007	0.0065
30-34	Male	0.0053	0.002	0.0006	0.0068
30-34	Female	0.0035	0.0027	0.0006	0.0071
35-39	Male	0.0049	0.0018	0.0007	0.0068
35-39	Female	0.003	0.0021	0.0008	0.0066
40-44	Male	0.0046	0.0017	0.0006	0.0061
40-44	Female	0.0027	0.0021	0.0005	0.0057
45-49	Male	0.0038	0.0017	0.0005	0.0052
45-49	Female	0.0021	0.0015	0.0005	0.0051
50-54	Male	0.0032	0.0012	0.0005	0.0051
50-54	Female	0.0016	0.0013	0.0005	0.0048
55-59	Male	0.0025	0.0013	0.0004	0.0048
55-59	Female	0.001	0.0013	0.0007	0.0044
60-64	Male	0.0018	0.0018	0.0004	0.0044
60-64	Female	0.0012	0.0009	0.0003	0.004
65-69	Male	0.0014	0.0014	0.0005	0.0037
65-69	Female	0.0011	0.0005	0.0005	0.0038
70-74	Male	0.001	0.001	0.001	0.0031
70-74	Female	0.0009	0.0009	0.0009	0.0045

[3]DPH Chapter 55 Data warehouse, datasets used include: APCD, BSAS, CASEMIX, DEATH, BIRTH, MATRIS, and PMPD

© 2023 Chatterjee A et al. *JAMA Network Open*.

75-79	Male	0.002	0.002	0.002	0.0039
75-79	Female	0.0013	0.0013	0.0013	0.0053
80-84	Male	0.0062	0.0031	0.0031	0.0062
80-84	Female	0.0026	0.0013	0.0013	0.0065
85-99	Male	0.0035	0.0035	0.0035	0.0069
85-99	Female	0.0011	0.0011	0.0011	0.0057

* Detox transition probabilities listed here needed to be calibrated to match observed (from MA PHD) detox admissions each year from 2013-2015. We calibrated time varying multipliers $\boldsymbol{\eta}(\boldsymbol{t})$on approximate rates $\tilde{\lambda}_{ND}$ to obtain accurate estimates for detox transition rates $\lambda_{ND}(t)$ where $t = 2013, 2014, 2015$. In other words, $\lambda_{ND}(t)$ are calculated as $\lambda_{ND}(t) = \tilde{\lambda}_{ND} \times \boldsymbol{\eta}(\boldsymbol{t})$.

B.1.4.2 Treatment Initiation Effect

When population begins a treatment for opioid use disorder, for example out-patient buprenorphine, a portion of the population immediately transitions from active to non-active use. Following that initial "treatment initiation effect" there is bidirectional movement between active and nonactive use states, even while engaged with treatment. The main source of data for the treatment initiation effect and for substance use transitions while engaged with buprenorphine, naltrexone, or methadone is the NIDA CTN urine toxicology data. The CTN trials collected routine periodic urine toxicology from all participants. While the published clinical trials results censored participants at the first relapse to drug use (the primary outcome of that trial), the trials continued to collect data from patients who experienced a relapse, such that the database includes longitudinal urine toxicology from patients who relapsed to active use, as well as some who remitted back to non-active use over the course of the trial. We analyzed those data in an "as treated" manner, such that RESPOND estimates realistic movements between active and non-active drug use states among people who are taking a medication. Note that relapsing to active drug use is not the same thing as loss to follow-up from treatment (see below).

Methodological Notes:

Upon entering treatment, a proportion of the population immediately transitions from active to non-active opioid use (***Supplemental Table 9***). This proportion $\hat{p}_{Init\_Act \rightarrow NonAct}$ is stratified by treatment episode as follows:

- buprenorphine (Bup): 0.74, based on the proportion of observed negative (non-active) urine samples at week 1
- naltrexone (Ntx): 0.90, based on the proportion of observed negative urine samples at week 5

© 2023 Chatterjee A et al. *JAMA Network Open*.

- methadone (Mmt): 0.57, based on the proportion of observed negative urine samples at week 5

We assume a binomial distribution and we use the Wald's method to calculate 95% CIs for the proportion $\hat{p}_{Act \rightarrow NonAct}$ representing the block initiation effect.

The model takes this input as the reciprocal, or $\hat{p}_{Act \rightarrow Act.}$ It is the direct reciprocal because the only transitions that can be made in this specific instance are Active to Non-active within the initial OUD type (injection vs. non-injection). There is no way to cross over to the other OUD type (ie there is no transition possible from Active non-injection to Non-active injection). These probabilities are listed below in Table 6. For example, take an individual who is in the 'Active non-injection' opioid use state and the 'No Treatment' care delivery state. When they move to the 'Buprenorphine' (Bup) care delivery state (based on their probability of movement identified in Table 5) they have a probability of 0.257 of **immediately** shifting to a 'Non-active non-injection' opioid use state. After that first week, their opioid use state transitions will instead be governed by the probabilities in **Supplemental Table 10**.

eTable 9. Treatment Initiation Effects Parameters: Immediate Transition Probabilities Modeling Movement Between OUD States When Movement Between Treatment States Occurs

**Initial OUD state***	**Probability of Staying in an Active Opioid Use State**			
	**Transition to Treatment (γ_T)**			**Transition to corresponding Post-treatment** [**] **(γ_P)**
	**Bup**	**Ntx**	**Mmt**	
Active non-injection	0.257(0.204, 0.309)	0.103(0.058, 0.148)	0.433(0.403, 0.462)	1
Active injection	0.257(0.204, 0.309)	0.103(0.058, 0.148)	0.433(0.403, 0.462)	1
Non-active non-injection	N/A***	N/A***	N/A***	N/A ****
Non-active injection	N/A***	N/A***	N/A***	N/A ****

* All individuals start in the 'No Treatment' care delivery state, since this is the only way to transition into a Treatment care delivery state

** These estimates are the same for post-Bup, post-Ntx, post-Mmt, and post-detox.

*** There is no block initiation effect for population that is not currently using opioids, because only population that is currently using opioids seeks care in the model.

**** Calibration parameter.

© 2023 Chatterjee A et al. *JAMA Network Open*.

B.1.4.3 Transitions Between Active and Non-Active Opioid Use While Engaged with Treatment

We estimated Weekly OUD transition probabilities $\hat{p}_{Trt\_Act \to NonAct}$ using Multi-State Models (MSMs) [15]. We fit separate models for each treatment: buprenorphine (Bup), naltrexone (Ntx), and methadone (Mmt), using data from the National Institute of Drug Abuse Clinical Trials Network (NIDA CTN) [4–6].

Structural Assumptions:

- Population engaged with treatment may move between active and non-active opioid use, but the population engaged with treatment does not change the route of administration of their opioid use. In other words, population that entered treatment using non-injection opioids will not escalate to injection drug use while still engaged with treatment (Core Simulation within OUD treatment episodes (blocks) – (*eFigure*).

Transition probabilities between active and non-active states are the same for both injection and non-injection drug use. This structural assumption is confirmed by the MSM estimates for buprenorphine and methadone models, in which route was included as a model covariate, but was not a significant predictor of transition rates

Methodological Notes:

- Each MSM includes age and sex as covariates.
- Age is included as a continuous covariate in the MSM model, thus allowing estimation of the transition probabilities for age bins in which data are not available. We consider five 5 age groups: 10–19, 20–24, 25–34, 35–49, and 50–99 years old.
- OUD transition for Buprenorphine and Methadone: We keep all the weekly MSM estimates of OUD transition probabilities except week 1, which is considered as block initiation.
- OUD transition for Naltrexone: We delete the estimates for the first 4 weeks due to the inaccurate results from detoxification. Week 5 is also excluded from the analysis, as it is considered as block initiation.
- Transition probabilities from non-active to active use are defined as: $\hat{p}_{Trt\_NonAct \to Act} = 1 - \hat{p}_{Trt\_Act \to NonAct}$

© 2023 Chatterjee A et al. *JAMA Network Open*.

eFigure 3. Core Simulation Within OUD Treatment Episodes (Blocks)

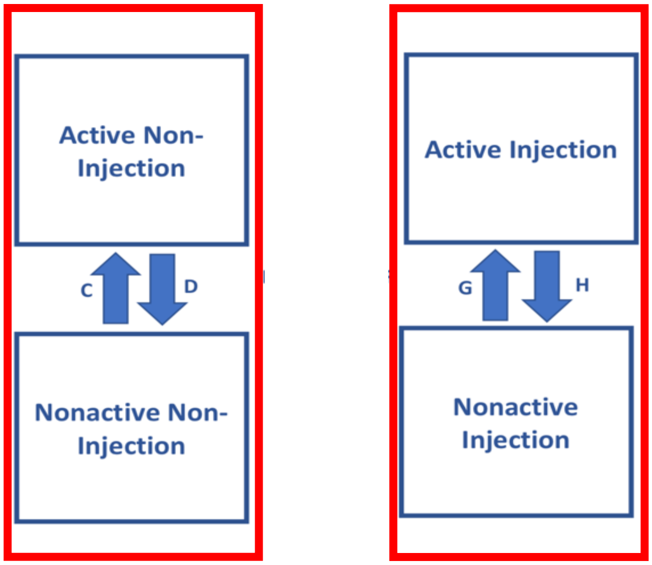

eTable 10. Transition Probabilities Between Active and Nonactive Opioid Use While Engaged With Treatment

Age	Sex	Initial OUD status*	Treatment		
			Buprenorphine	Naltrexone	Methadone
10-19	Male	Active	0.750	0.847	0.669
10-19	Female	Active	0.746	0.711	0.692
10-19	Male	Nonactive	0.156	0.107	0.112
10-19	Female	Nonactive	0.118	0.130	0.089
20-24	Male	Active	0.738	0.832	0.678
20-24	Female	Active	0.735	0.687	0.700
20-24	Male	Nonactive	0.148	0.099	0.120
20-24	Female	Nonactive	0.112	0.119	0.095
25-39	Male	Active	0.723	0.814	0.688
25-39	Female	Active	0.721	0.656	0.710
25-39	Male	Nonactive	0.139	0.090	0.128
25-39	Female	Nonactive	0.105	0.108	0.102
40-54	Male	Active	0.686	0.763	0.713
40-54	Female	Active	0.683	0.573	0.732
40-54	Male	Nonactive	0.119	0.072	0.152
40-54	Female	Nonactive	0.090	0.083	0.121
55-99	Male	Active	0.628	0.673	0.746

© 2023 Chatterjee A et al. *JAMA Network Open*.

55-99	Female	Active	0.626	0.443	0.762
55-99	Male	Nonactive	0.096	0.051	0.192
55-99	Female	Nonactive	0.072	0.056	0.154

* Probabilities listed here are transition probabilities to "Active" state. For ex:- when initial OUD status is "Active", probabilities listed here are probabilities of staying in the "Active" state.

B.1.4.4 Probability of Loss to Follow-Up

In every time step, the population that is engaged with treatment faces a risk of disengaging from care and being lost to follow-up. Loss to follow-up differs from relapse to active drug use while remaining engaged with opioid treatment. The population that disengages with care and is lost to follow-up enters the "post-treatment state," during which time they have a high rate of relapse to active use and a high rate of overdose among active users. The post-treatment block represents the period of time immediately following discontinuation of a medication or release from an abstinence-based setting (acute drug detoxification center, residential drug treatment, or jail), when opioid tolerance is low and the risk of overdose is higher than that of a person who never initiated treatment.

The main source of data for estimating the probability of loss to follow-up is Market Scan, a large insurance claims database containing millions of individuals who have commercial insurance coverage. As a randomized controlled trial, the CTN data cannot provide estimates of retention in care or loss to follow-up in the real world. We have previously published rates of loss to follow-up from buprenorphine and naltrexone treatment [16]. We therefore turn to Market Scan, which is nationally representative and reflects real-world practice in the U.S.

<u>Structural Assumptions:</u>

- In RESPOND, the only way to transition into a post-treatment episode is from a corresponding treatment episode.
- The "No Treatment" block does not have a post-treatment episode.
- RESPOND also considers the probability of immediate relapse to active opioid use upon being lost to follow-up from treatment:

Methodological Notes:

The weekly transition probability from treatment to post-treatment is calculated as:

$$\widehat{\mathbf{P}}_{\mathbf{Trt} \rightarrow \mathbf{Post-Trt\,.T}} = 1 - \exp\left(-\lambda_{TP}\right) \qquad (3)$$

where

$\lambda_{TP} = \frac{-log\{1-(1-p_T)\}}{t}$ and p_T: the retention probability for treatment T reported for the study time-period t in literature.

Supplemental Table 11 presents estimates of the weekly transition probabilities $\widehat{\mathrm{P}}_{\mathrm{Trt} \rightarrow \mathrm{Post-Trt\,.T}}$ based on data from Morgan et al, stratified by treatment [16].

eTable 11. Weekly Transition Probabilities From Treatment to Posttreatment

**Treatment *T***	p_T	Study time-period t	λ_{TP}	$\widehat{\mathbf{P}}_{\mathbf{Trt} \rightarrow \mathbf{Post-Trt\,.T}}$	**Source**
Bup	0.1760	52 weeks	0.0334	0.0328	Morgan et al. [16]
Naltrexone	0.0214	52 weeks	0.0739	0.0713	
Methadone	0.5240	20 weeks	0.0323	0.0318	Strain et al. [17]

B.1.5 Population Dynamics

B.1.5.1 Initial Cohort

To simulate the demography and OUD epidemiology in the underlying population, RESPOND requires the initial cohort to be specified as follows: 1) age and sex distributions of people with opioid use disorder, 2) proportion of people beginning in each drug-use state, and 3) proportion of people within each treatment episode.

Structural Assumptions:

- No population begins the simulation in a post-treatment block.
- RESPOND does not characterize the population by race or ethnicity.

Methodological Notes:

Supplemental Table 12 presents the key parameters related to cohort initialization.

© 2023 Chatterjee A et al. *JAMA Network Open*.

First, we took the estimates of the population of OUD between 2013 and 2015 from a age, sex, and county stratified capture-recapture analyses using the Massachusetts Public Health Data Warehouse (MA PHD) [7]. The capture-recapture approach provides a method to estimate the total population with high-risk opioid use in a given calendar year, including those who have not been identified as being an opioid user and do not appear in medical claims or prevalence surveys. The previous work in provides estimates for year 2012, however, as it has been noted in the paper, the data sources used before 2013 were not complete [7]. Hence, we used data in the years with more comprehensive data sources to predict the data in 2012 which has fewer data sources.

Second, we obtained the age and sex stratified counts for alive OUD population at the end of each year by subtracting death with opioid overdose involved from the estimated total size.

Then, we applied a Negative Binomial regression on the alive counts to predict the counts in the end of 2012, with age, sex and calendar years being the covariates. An interaction of Age groups and sex was applied in the model. We treated this predicted count as the alive population size in the end of year 2012 and assumed that it constructs the initial cohort beginning in year 2013. To further identify the number of OUD in no treatment, as well as in treatment, at the beginning in year 2013, we identified OUD as we did in Barocas et al. and then counted the number of OUD in treatment (N_0) in January 2013. The remaining population size is called "the number of OUD in no treatment"(T_0) [7].

eTable 12. Initializing Cohort Parameters

Parameter	Value	Method	Years	Stratification	Time Varying	Source
Total ($\hat{N}_{OUD,t}$)	-	Capture recapture	2013-2015	Age (3 groups*) & Sex	Yes	MA PHD analysis update to Barocas et al. [7]
By age-group	-	Observed	2012-2015	Age (18 groups**) & Sex	Yes	US Census 2010
Proportion with injection drug use	25.09%	Observed	2013	Age (3 groups*) & Sex	No	NSDUH
Proportion non-actively using	9%	Estimated	-	-	No	CDC [8]<br>Cedarbaum et al [9]

Abbreviations:
- MA DPH: Massachusetts Department of Public Health data
- NSDUH: National Survey on Drug Use and Health

* 3 age groups: 10 – 24, 25 – 44, 45 – 99
** 18 age groups: 5-year age-groups from 10-99

© 2023 Chatterjee A et al. *JAMA Network Open*.

B.1.5.2 Aging

RESPOND simulates discrete time steps (rather than continuous time) and categorical age groups or "brackets" over the lifetime. The user can define the bounds of age groups so as to match the structure of the underlying population. Aging occurs as the population progresses to the next age group after a number of cycles that is determined by the size of the age brackets. The model employs a half-cycle correction and aging occurs in discrete steps, namely only at multiples of the age group size.

Structural Assumptions:

- The entire population of the last age bracket (95 to 100-year-olds) is removed from the simulation at each aging cycle and replaced by the population from the previous age bracket.

B.1.5.3 Entering Cohort (New OUD Arrivals)

RESPOND can simulate either an open cohort (meaning that new individuals arrive to the population over the course of the simulation), or a closed cohort. Here we consider an open cohort simulation of OUD population in state of Massachusetts. When running the model to simulate an open cohort, arrivals to the population occur at every time step. Arrival rates are stratified by age and sex and vary over time to realistically reflect the incidence of new opioid use disorder and/or movement into the jurisdiction.

Structural Assumptions:

- All new populations enter the first block ("No Treatment" episode) and the first OUD state (currently active, non-injection).
- All new arrivals enter the simulation as active non-injectors under the "No Treatment" block but can transition to other feasible OUD states in the OUD transition module.

Methodological Notes:

Supplemental Table 13 and **Supplemental Table 14** present the key parameters related to entering cohort.

- The size of the entering cohort ($N_{enter,t}$) each year defined as:

$$N_{enter,t} = \widehat{N}_{OUD,t} - (\widehat{N}_{OUD,t-1} - \widehat{N}_{FOD,t-1} - \widehat{N}_{Non\text{-}FOD,t-1}) \qquad (1)$$

where $\widehat{N}_{OUD,t}$ is the estimated OUD population in year t, $\widehat{N}_{FOD,t-1}$ is the estimated number of fatal overdoses, and $\widehat{N}_{Non\_FOD,t}$ is the estimated total number of deaths from other causes, in the previous year (t-1).

© 2023 Chatterjee A et al. *JAMA Network Open*.

- Let $\alpha_{j,k}(t)$ be age-sex stratified weekly new OUD arrival count in year t, where j, and k represent age and sex strata respectively and t=2013, 2014, 2015. Then $\alpha_{j,k}(\text{t}) = \mathbf{A}(\mathbf{t}) \times p_{j,k}(\text{t})$. Here $\mathbf{A}(\mathbf{t})$ is the weekly non-stratified total arrivals and $p_{j,k}(\text{t})$ denotes yearly time varying demographic proportions. We calculate $\mathbf{A}(\mathbf{t})$ from $N_{enter,t}$ as $\mathbf{A}(\mathbf{t}) = N_{enter,t}/52$.

eTable 13. Entering Cohort Parameters

Parameter	Description	Value	Method	Stratification	Source
$N_{initial}$	Initial cohort size combining no treatment size and treatment size	215,918	Estimated from NB regression	Age (j=1,..,3 groups*)<br>Sex (k=1,2)<br>Time (t=2013, 14, 15)	MA PHD Repository<br>Barocas et al. [7]
$N_{enter,t}$	Entering cohort size	$\widehat{N}_{OUD,t} - \widehat{N}_{initial,2013}$	Calculated, See Table 3	Time (t=2013)	
$N_{enter,t}$		$\widehat{N}_{OUD,t} - (\widehat{N}_{OUD,t-1} - \widehat{N}_{FOD,t-1} - \widehat{N}_{Non\text{-}FOD,t-1})$	Calculated, See Table 3	Time (t=2014, 15)	
$\widehat{N}_{FOD,t}$	Fatal overdoses	Varies	Estimated	Time (t=2013, 14, 15)	
$\widehat{N}_{Non\text{-}FOD,t}$	Non-FOD deaths	Varies	Estimated	Age (j=1,..,5 groups**)<br>Sex (k=1,2)	MA PHD[3]
$p_{jk}(t)$	Entering cohort demographic proportions at each cycle (week)	Varies	Observed Imputation of missing strata %	Age (j=1,..,5 groups**)<br>Sex (k=1,2)<br>Time (t=2013, 14, 15)	NSDUH MRB Statistical Inference Report [10]

Abbreviations:
- MA DPH: Massachusetts Department of Public Health data
- NSDUH: National Survey on Drug Use and Health
- MRB: Methodological Resource Book
- NB: Negative Binomial Distribution

* 3 age groups: 10 – 24, 25 – 44, 45 – 99
** 5 age groups: 10 – 19, 20 – 24, 25 – 34, 35 – 49, 50 – 99
*** Other cause mortality is from Chapter 55 assuming that is no additional death in one year.

eTable 14. Entering Cohort Counts by Year

Total counts (population size)	Year			
	2012***	2013	2014	2015
Population of OUD at time t ($\widehat{N}_{OUD,t}$)	166,835	226,861 (189832,301264)	233,184 (178671,271814)	275,070 (222160,357383)
Total number of non-FOD*deaths ($\widehat{N}_{D\_other,t}$)	596	964	1,332	1,605
Total number of FOD* deaths ($\widehat{N}_{FOD,t}$)	702	900	1,294	1,562

© 2023 Chatterjee A et al. *JAMA Network Open*.

Entering Cohort (yearly total) ($N_{enter,t}$)	**-**	**10,943**	**8,187**	**44,512**
Entering Cohort (Weekly total) ($\mathbf{A(t)}$)	**-**	**210**	**157**	**856**
*** The estimates and observed data in year 2012 do not include data from Emergency Medical Services (EMS). Subsequent years do include those data.				

© 2023 Chatterjee A et al. *JAMA Network Open*.

C. COSTS

eTable 15. Cost of Naltrexone Treatment for Opioid Use Disorder

	Non-facility total	Facility total	Conversion factor	Cost-NonFac	Cost- Fac	Annual Cost-Nonfac	Annual Cost - fac	Weekly Cost-nonfac
**Quarterly MD visit**								
**Visit New**								
**Level 3 [CPT 99203]**	3.05	2.15	36.0391	109.919255	77.484065	54.96	38.7420325	
**Level 4 [CPT 99204]**	4.63	3.64	36.0391	166.861033	131.182324	83.43	65.591162	
**Visit Established**								
**Level 3 [CPT 99213]**	2.09	1.44	36.0391	75.321719	51.896304	112.98	77.844456	
**Level 4 [CPT 99214]**	3.06	2.22	36.0391	110.279646	80.006802	165.42	120.010203	
**Monthly Nurse visit [CPT 99211]**	0.64	0.26	36.0391	23.065024	9.370166	184.52	74.961328	
**Monthly depot injection (CPT code 96327)**				16.94	16.94	203.28	203.28	
**Annual HIV test [CPT 86701]**				9.87	9.87	9.87	00009.87	
**Annual Hepatitis A/B/C test [CPT 86704, 86706, 86708, 86803, 87340]**				66.41		66.41		
**Annual syphillis blood test [CPT 86593]**				00004.89	00004.89	4.89	00004.89	
**Annual gonorrhea/chlamydia test [CPT 87491, 87591]**				77.98		77.98		
**Monthly urine toxicology screen (CPT code 80306)**				00017.14	00017.14	205.68	205.68	

© 2023 Chatterjee A et al. *JAMA Network Open*.

**Monthly cost of Vivitrol itself [in drug costs]**			
	**Total Cost:**	1169.42	24.36296632

eTable 16. Cost of Buprenorphine Treatment for Opioid Use Disorder

	Non-facility total	Facility total	Conversion factor	Cost-NonFac	Cost- Fac	Annual Cost-Nonfac	Annual Cost fac	Weekly Cost-nonfac
**Monthly MD visit**								
**Visit New**								
**Level 3 [CPT 99203]**	3.05	2.15	36.0391	109.919255	77.484065	54.96	38.7420325	
**Level 4 [CPT 99204]**	4.63	3.64	36.0391	166.861033	131.182324	83.43	65.591162	
**Visit Established**								
**Level 3 [CPT 99213]**	2.09	1.44	36.0391	75.321719	51.896304	414.27	285.429672	
**Level 4 [CPT 99214]**	3.06	2.22	36.0391	110.279646	80.006802	606.54	440.037411	
**Weekly Nurse visit [CPT 99211]**	0.64	0.26	36.0391	23.065024	9.370166	922.60	374.80664	
**Annual HIV test [CPT 86701]**				00009.87	00009.87	9.87	00009.87	
**Annual Hepatitis A/B/C test [CPT 86704, 86706, 86708, 86803, 87340]**				66.41		66.41		
**Annual syphillis blood test [CPT 86593]**				00004.89	00004.89	4.89	00004.89	

© 2023 Chatterjee A et al. *JAMA Network Open*.

**Annual gonorrhea/chlamydia test [CPT 87491, 87591]**	77.98		77.98		
**Weekly urine toxicology screen [CPT code 80306]**	00017.14	00017.14	891.28	891.28	
		**Total Cost:**	3132.2<br>3		65.2547627<br>4

*CPT Codes found through the American Medical Association [21]

© 2023 Chatterjee A et al. *JAMA Network Open*.

eTable 17. Cost of Methadone Treatment for Opioid Use Disorder

Annual Cost	Daily Cost	Weekly Cost	Weekly cost minus medication	Source
6552		126	121.69	NIDA [22]

eTable 18. Cost of Nonfatal Overdose

	Emergency Room	Inpatient
**Proportion** [23]	60%	40%
**Cost**	$962.48 (2019 dollars) [24,25]	$9,120 (2019 dollars) [23]
**Proportion * Cost**	0.6 * 962.58	0.4 * 9120
**Subtotals**	$577.55	$3,648
**Average Treatment Cost** Sum of Emergency Room + Inpatient	4,225.55	
**Ambulance Cost** [26]	$331.10	
**Total Cost** Treatment Cost + Ambulance Cost	**$4556.65**	

Description: Non-fatal overdose costs: Of 2014 overdoses in RI ~60% went to the ER only and 40% were then inpatient [23]. Cost for an ER stay was $908 in 2016 dollars, which is $962.48 in 2019 dollars [24,25]. Total costs (not charges) of all inpatient treatment for OUD was $13,799,794 (in 2019 dollars) which was approximately $9120 per person [23]. This means for ~60% of all overdoses, the cost was ED only and for 40% of overdoses the cost was inpatient costs. This gives an equation of 0.6*928.48 + 0.4*9120.82. In addition, we add cost of ambulance to everybody, which is an additional $331.10 (converted to 2019 dollars) [26].

eTable 19. Cost of Fatal Overdose

Fatal OD Cost				
MA DPH data				
	Counts	Deaths	Percent total fatal OD	Cost of fatal OD
Fatal OD (2013-2015) [23]	3756	3756		
All type opioid OD (2013-2015) [23]	29167			
ER	17500.2	52.5006	0.013977796	962.48
Inpatient	11666.8	233.336	0.062123536	9120.82
Unobserved fatal OD		3470.1634	0.923898669	331.1
Cost of fatal OD				885.97

Description: Fatal overdose: Using MA PHD data, 2013-2015 MA had 29,167 all types overdoses and 3,756 fatal overdoses. Some of the non-fatal overdoses would likely not have been observed, but this is

difficult to account for. To attempt to do this, we used death percentages from Jiang et al to predict how many of the all-types overdose deaths would likely have happened in a hospital setting [23].

Of 29,167 deaths, we assume 60% (17,500) were observed in the ED and 40% (11,667) inpatient (ratios from Jiang et al paper). Jiang et al had a 1.4% fatal OD rate in the ED and 6.2% fatal OD rate inpatient. Using our numbers, this means we could expect 53 fatal overdoses in the ED and 233 inpatient. This leaves 3,470 fatal overdoses unaccounted for (unobserved by the healthcare sector). So- of 3,756 fatal overdoses, 53 are assigned a cost of ED only, 233 are assigned cost of inpatient, and 3,470 are assigned cost of ambulance only (we assume all fatal overdoses, even those dead on ambulance arrival, would incur an ambulance cost) [26]. With that in mind, fatal overdose cost comes to $885.97.

© 2023 Chatterjee A et al. *JAMA Network Open*.

D. CORRECTIONS-SPECIFIC SUPPLEMENTAL TABLES AND FIGURES

eTable 20. 1- and 5-Year MOUD Initiations (Closed Cohort)

Treatment	No Intervention	XR-NTX Only (66% initiation)	All three MOUD (66% initiation)
1-year			
Buprenorphine (95% UI)	5,973 (5,871, 6,034)	5,250 (5,127, 5,324)	12,305 (11,899, 12,701)
Naltrexone (95% UI)	686 (675, 692)	9,981 (9,487, 10,522)	638 (624, 647)
Methadone (95% UI)	1,755 (1,725, 1,773)	1,543 (1,507, 1,565)	5,304 (5,092, 5,517)
**Total (95% UI)**	**8,414 (8,271, 8,499)**	**16,774 (16,285, 17,241)**	**18,247 (17,824, 18,639)**
5-year			
Buprenorphine (95% UI)	29,163 (27,786, 29,990)	28,075 (26,795, 28,878)	36,794 (35,455, 37,728)
Naltrexone (95% UI)	3,184 (3,047, 3,267)	15,054 (14,327, 15,782)	3,077 (2,947, 3,157)
Methadone (95% UI)	8,580 (8,175, 8,827)	8,264 (7,885, 8,503)	12,979 (12,480, 13,353)
**Total (95% UI)**	**40,927 (39,001, 42,082)**	**51,393 (49,438, 52,714)**	**52,850 (50,962, 54,167)**

© 2023 Chatterjee A et al. *JAMA Network Open*.

eTable 21. Percent Retained on MOUD at 6 Months Post Release (Closed Cohort)

	XR-NTX Only (66% initiation)	All three MOUD (66% initiation)
Total cohort	30,000	30,000
# who link to care post-release	8,908 (30%)	11,037 (37%)
# retained at 6 months	1,915 (6% of total cohort; 21% of those linked post-release)	4,517 (15% of total cohort; 41% of those linked post-release)

eTable 22. 1- and 5-Year Treatment Starts (MA Population Open Cohort)

Treatment	No Intervention	XR-NTX Only (66% initiation)	All three MOUD (66% initiation)
1-year			
Buprenorphine (95% UI)	56,212 (52,028, 58,125)	55,863 (51,982, 57,801)	60,810 (56,521, 62,851)
Naltrexone (95% UI)	7,120 (6,688, 7,335)	13,444 (12,712, 13,915)	7,120 (6,686, 7,338)
Methadone (95% UI)	15,847 (14,615, 16,411)	15,753 (14,624, 16,325)	18,359 (17,063, 18,963)
**Total (95% UI)**	**79,179 (73,339, 81,872)**	**85,060 (79,492, 87,889)**	**86,289 (80,305, 89,036)**
5-year			
Buprenorphine (95% UI)	296,438 (272,795, 307,292)	293,896 (271,811, 305,162)	317,448 (293,371, 329,317)
Naltrexone (95% UI)	40,608 (38,031, 41,910)	72,210 (68,158, 74,855)	40,373 (37,820, 41,695)
Methadone (95% UI)	83,983 (77,033, 87,276)	83,293 (76,853, 86,618)	95,984 (88,674, 99,502)
**Total (95% UI)**	**421,029 (387,698, 4346,523)**	**449,399 (417,686, 465,660)**	**453,805 (420,161, 470,063)**

© 2023 Chatterjee A et al. *JAMA Network Open*.

eTable 23. Undiscounted Budgetary Impact for MA Over 5 Years of Intervention (2021-2025), Total Cost in Billions of USD

Cost	No Intervention	XR-NTX Only (66% initiation)	All three MOUD (66% initiation)
Overdose[1] (95% UI)	0.31 (0.18, 0.51)	0.31 (0.18, 0.50)	0.31 (0.18, 0.50)
Healthcare utilization[2] (95% UI)	26.17 (24.24, 27.90)	26.13 (24.28, 27.51)	26.04 (24.13, 27.75)
OUD treatment[3] (95% UI)	2.30 (2.04, 2.80)	2.52 (2,19, 2.93)	2.45 (2.06, 2.86)
Incarceration (95% UI)	0.61 (0.59, 0.62)	0.60 (0.58, 0.61)	0.60 (0.58, 0.61)
Health Care Sector Perspective Total[4] (95% UI)	28.79 (26.94, 30.70)	28.96 (26.95, 30.94)	28.80 (26.03, 30.94)
Modified Societal Perspective Total[5] (95% UI)	29.40 (27.53, 31.32)	29.56 (27.53, 31.55)	29.41 (26.61, 31.55)

1. Overdose costs include the cost of both fatal and non-fatal overdoses.
2. Healthcare utilization costs include all healthcare costs outside of MOUD treatment and overdose costs.
3. OUD treatment costs include all MOUD treatment-related costs, including pharmaceutical costs and treatment utilization costs such as doctor visits, laboratory testing, and procedure costs.
4. Health care sector perspective includes the cost of overdose, healthcare utilization, and OUD treatment.
5. Modified societal perspective includes cost of overdose, healthcare utilization, OUD treatment, and incarceration.

eTable 24. Sensitivity Analysis Using Multiplicative Approach: ICER Table

Intervention	Discounted Cost Per Person	Change in Discounted Cost Per Person	Discounted QALY Per Person	Change in Discounted QALY Per Person	ICER

© 2023 Chatterjee A et al. *JAMA Network Open*.

No Intervention (reference)<br>(95% UI)	$302,781 ($289,649, $321,891)	-	9.96 (9.57, 10.53)	-	-
MOUD intervention, All three MOUD, 66%<br>(95% UI)	$303,633 ($290,268, $321,273)	$851	10.06 (9.67, 10.61)	0.10	$7,962
MOUD intervention, XR-NTX Only, 66%<br>(95% UI)	$306,356 ($291,143, $322,100)	$2,723	10.03 (9.60, 10.56)	-0.03	**Dominated**

eTable 25. Deterministic Sensitivity Analyses

Parameter	Baseline Value	Range	All three MOUD ICER evaluated at lower and upper bound of range
Proportion Male	0.68	0.54 – 0.72	$6,881 - $7,573
Mean Age	37	27 - 47	$7,252 - $7,252
Proportion with injection drug use at baseline	0.25	0.2 – 0.3	$6,791 - $7,709
Proportion actively using at baseline	0.84	0.67 - 1	$6,477 - $8,177
SMR- injection	5.1	4.4 – 5.7	$7,182 - $7,314
SMR- non-injection	2.1	1.8 – 2.3	$7,079 - $7,452
Movement into corrections	7.8	1.2 – 26.4	$5,875 - $9,911
Movement onto Buprenorphine	19.9	11.4 – 41.8	$6,615 - $8,862
Movement onto Methadone	5.9	2.4 – 24.4	$7,053 - $8,260
Movement onto Naltrexone	2.3	0.7 – 21.5	$5,422 - $7,406
6-month Buprenorphine retention	0.34	0.17 – 0.54	$6,231 - $8,165
6-month Methadone retention	0.55	0.28 – 0.84	$6,699 - $7,530
6-month Naltrexone retention	0.21	0.11 – 0.32	$7,202 - $7,292
Monthly overdose rate- No Treatment	6.75	6 – 11.2	$6,539 - $10,470

© 2023 Chatterjee A et al. *JAMA Network Open*.

Monthly overdose rate- Buprenorphine	2.73	2.2 – 4.4	$7,039 - $7,323
Monthly overdose rate- Naltrexone	5.83	4.8 – 9.2	$7,251 - $7,257
Monthly overdose rate- Methadone	5.07	4.4 – 8.4	$6,774 - $7,344
Monthly overdose rate- Corrections	3.46	3 – 5.6	$7,173 - $7,653
Monthly overdose rate Post-Treatment	12.95	11.4 – 21.6	$6,377 - $7,402
Fatal OD proportion	0.137	0.13 – 0.13	$6,815 - $7,959
Treatment cost- Detox	2863	805 - 8204	$3,251 - $8,794
Treatment cost- Buprenorphine	114	75 - 163	$4,057 - $11,276
Treatment cost- Methadone	128	116 - 136	$6,284 - $7,933
Treatment cost- Naltrexone	327	316 - 338	$7,246 - $7,258
Treatment cost- Corrections Buprenorphine	115	92 - 138	$6,443 - $7,992
Treatment cost- Corrections Methadone	126	116 - 136	$6,918 - $7,517
Treatment Cost- Corrections Naltrexone	250	200- 300	$6,790 - $7,644
Fatal OD cost	858	443 - 1329	$7,235 - $7,268
Non-fatal OD cost	4557	2279 - 6836	$6,727 - $7,778

Note: The XR-NTX only strategy was dominated in all deterministic sensitivity analyses.

© 2023 Chatterjee A et al. *JAMA Network Open*.

eFigure 4. Overdose Deaths Among MA Population During 5-Year Intervention with Varying Initiation of XR-NTX Strategy

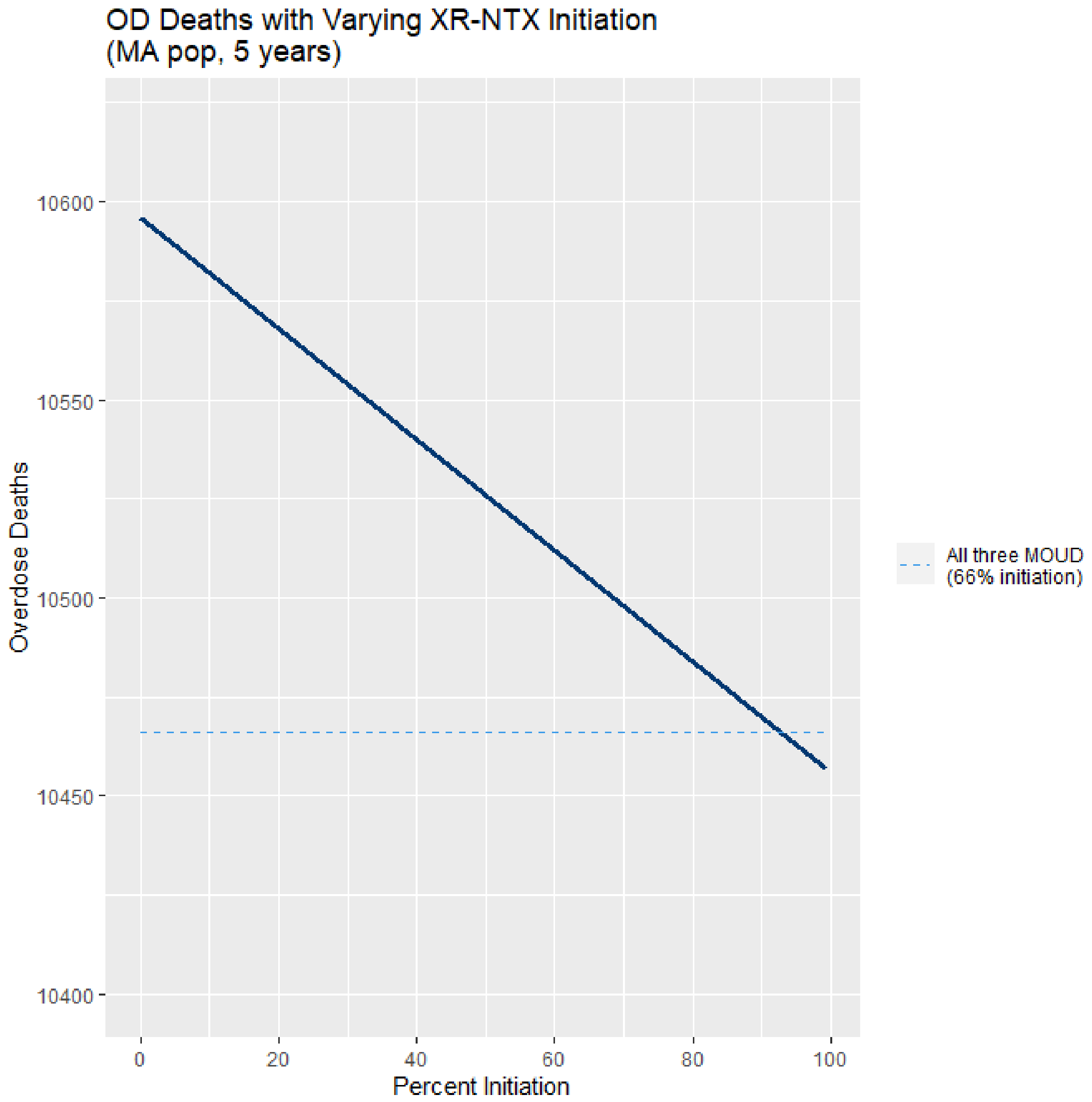

When compared to the all three MOUD strategy with 66% initiation, offering XR-NTX only does not result in fewer overdose deaths over the 5-year intervention period unless XR-NTX initiation is greater than 93%. In the hypothetical (extreme) scenario of 93% initiation on the XR-NTX strategy, the model estimates 10,465 overdose deaths over the 5-year intervention period compared to 10,466 overdose deaths from the all three MOUD strategy.

© 2023 Chatterjee A et al. *JAMA Network Open*.

eFigure 5. Fatal Overdoses Averted, Compared With No Intervention (A) A Closed Cohort of 30,000 Incarcerated Individuals in MA, Receiving the Intervention at the Beginning of the 5-year Period, and B) An Open Cohort Simulating the Population of the Entire State

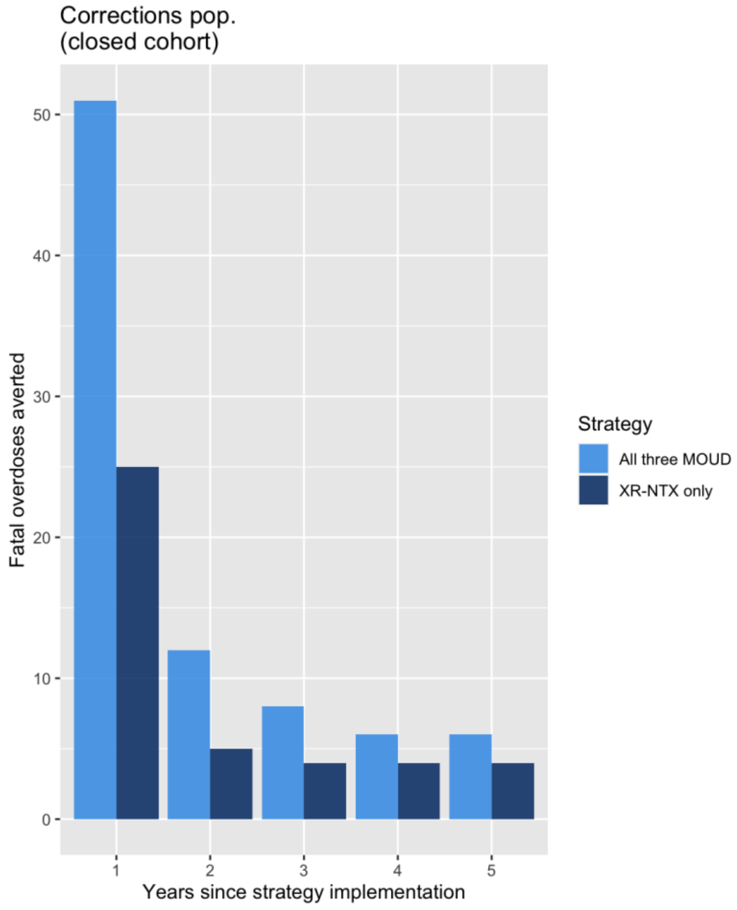

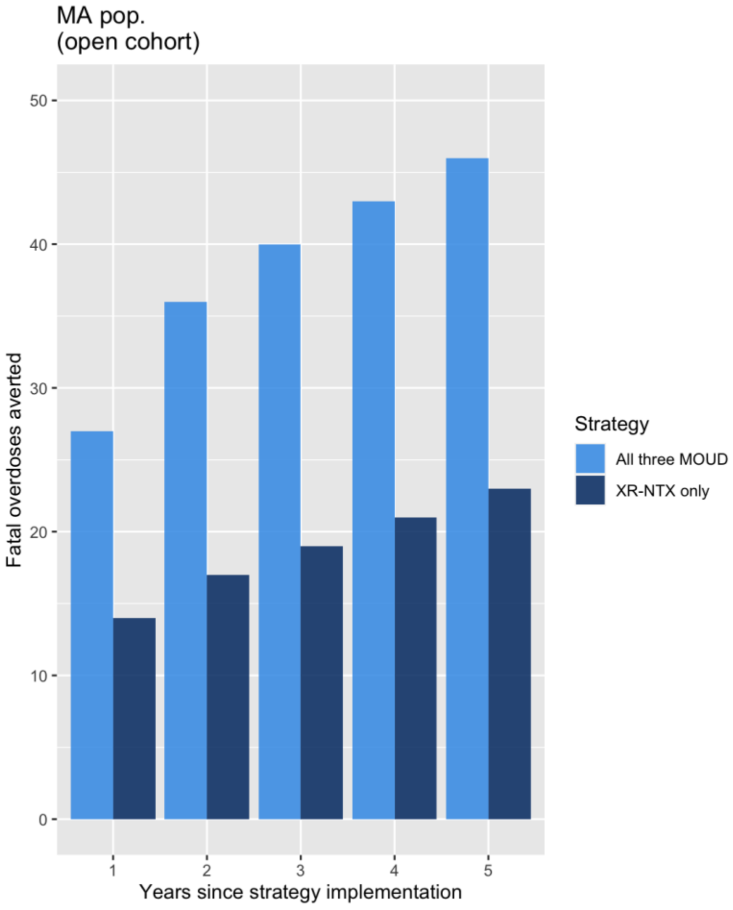

© 2023 Chatterjee A et al. *JAMA Network Open*.

eFigure 6. Cost-effectiveness Acceptability Curve (CEAC) of Tested Strategies

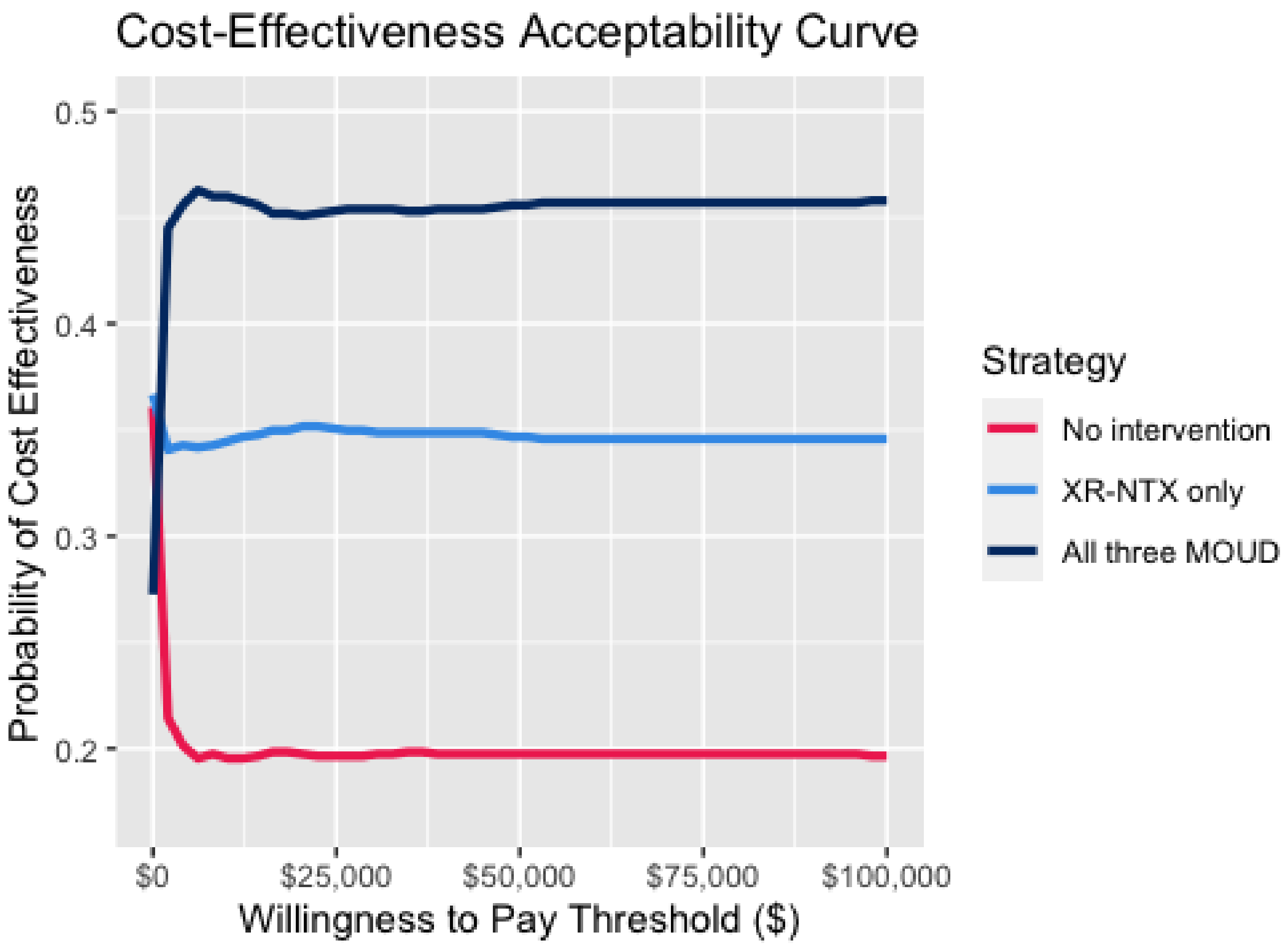

© 2023 Chatterjee A et al. *JAMA Network Open*.

E. CALIBRATION

Supplemental Figure 7 demonstrates the results of the validation of the extension of the model using a target of fatal overdose count. Observed fatal overdoses are found in the Medical Examiner's 2022 Data Brief on Opioid-Related Overdose Deaths among Massachusetts Residents [20]. Upper and lower confidence intervals (CI) were obtained by calculating a 10% difference in the observed value and adding (upper CI) and subtracting (lower CI) that value from the observed. With the exception of 2013, the model values are within the set confidence intervals and track observed fatal overdose values closely between 2014 and 2021.

eFigure 7. Fatal Overdoses as a Model Calibration Target

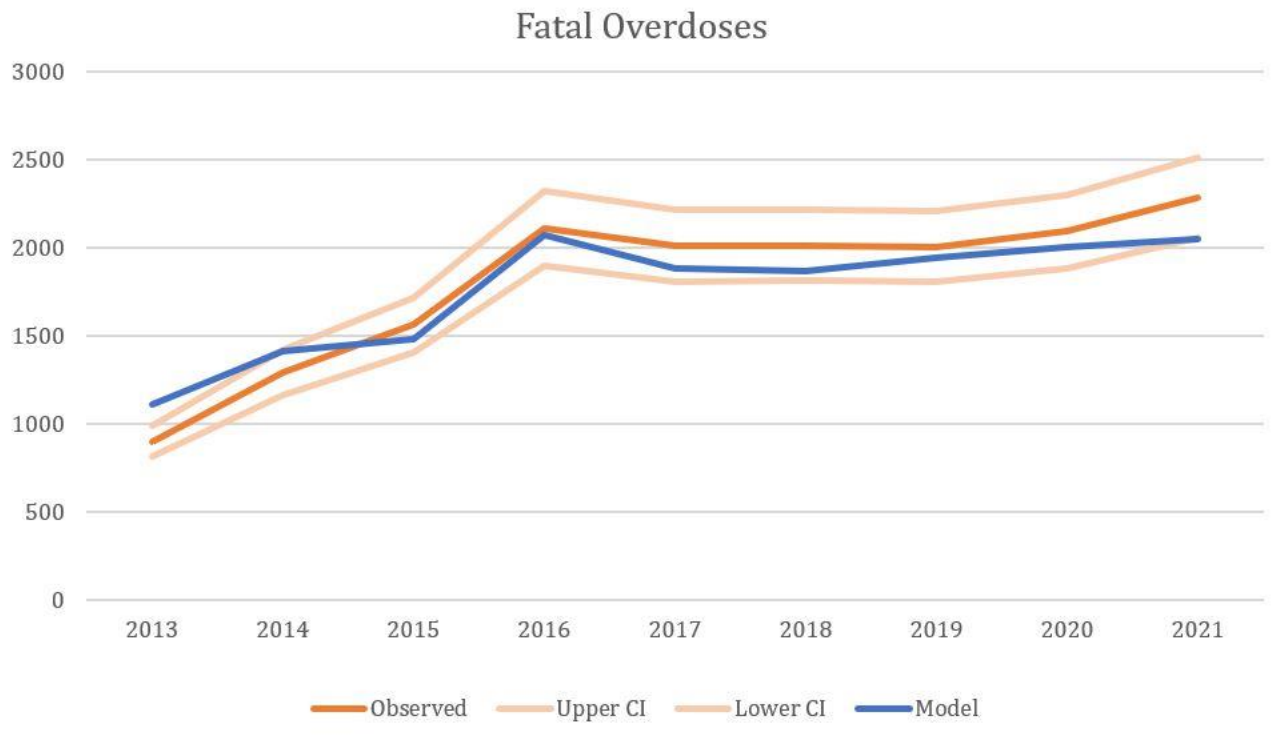

© 2023 Chatterjee A et al. *JAMA Network Open*.

F. REFERENCES

eReferences

1. Siebert U, Alagoz O, Bayoumi AM, et al. State-Transition Modeling: A Report of the ISPOR-SMDM Modeling Good Research Practices Task Force-3. *Value in Health*. 2012;15(6):812-820. doi:10.1016/j.jval.2012.06.014

2. Sonnenberg FA, Beck JR. Markov-Models in Medical Decision-Making - a Practical Guide. *Med Decis Making*. 1993;13(4):322-338. doi:10.1177/0272989x9301300409

3. Public Health Data Warehouse (PHD) | Mass.gov. Accessed January 11, 2023. https://www.mass.gov/public-health-data-warehouse-phd

4. Lee JD, Nunes EV Jr, Novo P, et al. Comparative effectiveness of extended-release naltrexone versus buprenorphine-naloxone for opioid relapse prevention (X:BOT): a multicentre, open-label, randomised controlled trial. *Lancet*. 2018;391(10118):309-318. doi:10.1016/S0140-6736(17)32812-X

5. Lee JD, Nunes EV, Mpa PN, et al. NIDA Clinical Trials Network CTN-0051, Extended-Release Naltrexone vs. Buprenorphine for Opioid Treatment (X:BOT): Study design and rationale. *Contemp Clin Trials*. 2016;50:253-264. doi:10.1016/j.cct.2016.08.004

6. Nunes EV, Lee JD, Sisti D, et al. Ethical and clinical safety considerations in the design of an effectiveness trial: A comparison of buprenorphine versus naltrexone treatment for opioid dependence. *Contemp Clin Trials*. 2016;51:34-43. doi:10.1016/j.cct.2016.09.006

7. Barocas JA, White LF, Wang JN, et al. Estimated Prevalence of Opioid Use Disorder in Massachusetts, 2011-2015: A Capture-Recapture Analysis. *Am J Public Health*. 2018;108(12):1675-1681. doi:10.2105/Ajph.2018.304673

8. HIV Surveillance | Reports| Resource Library | HIV/AIDS | CDC. Published January 30, 2023. Accessed February 6, 2023. https://www.cdc.gov/hiv/library/reports/hiv-surveillance.html

9. Cedarbaum ER, Banta-Green CJ. Health behaviors of young adult heroin injectors in the Seattle area. *Drug and Alcohol Dependence*. 2016;158:102-109. doi:10.1016/j.drugalcdep.2015.11.011

10. Substance Abuse and Mental Health Services Administration R MD. 2015 National Survey on Drug Use and Health: Methodological Resource Book. *Center for Behavioral Health Statistics and Quality*. 2017;(Section 13, Statistical Inference Report).

11. Neaigus A, Gyarmathy VA, Miller M, Frajzyngier VM, Friedman SR, Des Jarlais DC. Transitions to Injecting Drug Use Among Noninjecting Heroin Users: Social Network Influence and Individual Susceptibility. *JAIDS Journal of Acquired Immune Deficiency Syndromes*. 2006;41(4):493-503. doi:10.1097/01.qai.0000186391.49205.3b

© 2023 Chatterjee A et al. *JAMA Network Open*.

12. Shah NG, Galai N, Celentano DD, Vlahov D, Strathdee SA. Longitudinal predictors of injection cessation and subsequent relapse among a cohort of injection drug users in Baltimore, MD, 1988-2000. *Drug Alcohol Depend*. 2006;83(2):147-156. doi:10.1016/j.drugalcdep.2005.11.007

13. Nosyk B, Li L, Evans E, et al. Characterizing longitudinal health state transitions among heroin, cocaine, and methamphetamine users. *Drug Alcohol Depend*. 2014;140:69-77. doi:10.1016/j.drugalcdep.2014.03.029

14. Bailey GL, Herman DS, Stein MD. Perceived relapse risk and desire for medication assisted treatment among persons seeking inpatient opiate detoxification. *J Subst Abuse Treat*. 2013;45(3):302-305. doi:10.1016/j.jsat.2013.04.002

15. Jackson C. Multi-State Models for Panel Data: The msm Package for R. *2011*. 2011;38(8):28. doi:10.18637/jss.v038.i08

16. Morgan JR, Schackman BR, Leff JA, Linas BP, Walley AY. Injectable naltrexone, oral naltrexone, and buprenorphine utilization and discontinuation among individuals treated for opioid use disorder in a United States commercially insured population. *J Subst Abuse Treat*. 2018;85:90-96. doi:10.1016/j.jsat.2017.07.001

17. Strain EC, Stitzer ML, Liebson IA, Bigelow GE. Dose-response effects of methadone in the treatment of opioid dependence. *Annals of Internal Medicine*. 1993;119(1):23-27.

18. Sordo L, Barrio G, Bravo MJ, et al. Mortality risk during and after opioid substitution treatment: systematic review and meta-analysis of cohort studies. *BMJ*. 2017;357:j1550. doi:10.1136/bmj.j1550

19. NVSS - National Vital Statistics System Homepage. Published January 30, 2023. Accessed February 3, 2023. https://www.cdc.gov/nchs/nvss/index.htm

20. Massachusetts Department of Public Health. Data Brief: Opioid-Related Overdose Deaths among Massachusetts Residents. Published June 2022. Accessed December 28, 2022. https://www.mass.gov/doc/opioid-related-overdose-deaths-among-ma-residents-june-2022/download

21. CPT® Codes. American Medical Association. Published February 2, 2023. Accessed February 6, 2023. https://www.ama-assn.org/topics/cpt-codes

22. National Institute on Drug Abuse. How much does opioid treatment cost? National Institute on Drug Abuse. Published --. Accessed February 6, 2023. https://nida.nih.gov/publications/research-reports/medications-to-treat-opioid-addiction/how-much-does-opioid-treatment-cost

23. Jiang Y, McDonald JV, Koziol J, McCormick M, Viner-Brown S, Alexander-Scott N. Can Emergency Department, Hospital Discharge, and Death Data Be Used to Monitor Burden of Drug Overdose in Rhode Island? *Journal of Public Health Management and Practice*. 2017;23(5):499-506. doi:10.1097/PHH.0000000000000514

24. Medical Expenditure Panel Survey Home. Accessed February 6, 2023. https://meps.ahrq.gov/mepsweb/index.jsp

© 2023 Chatterjee A et al. *JAMA Network Open*.

25. Murphy SM, McCollister KE, Leff JA, et al. Cost-Effectiveness of Buprenorphine–Naloxone Versus Extended-Release Naltrexone to Prevent Opioid Relapse. *Ann Intern Med*. 2019;170(2):90-98. doi:10.7326/M18-0227

26. Coffin PO, Sullivan SD. Cost-effectiveness of distributing naloxone to heroin users for lay overdose reversal. *Ann Intern Med*. 2013;158(1):1-9. doi:10.7326/0003-4819-158-1-201301010-00003

© 2023 Chatterjee A et al. *JAMA Network Open*.
