# Supplementary material for: Estimated Costs and Outcomes Associated With Use and Nonuse of Medications for Opioid Use Disorder During Incarceration and at Release in Massachusetts
Source: JAMA Netw Open. 2023 Apr 14;6(4):e237036. doi: 10.1001/jamanetworkopen.2023.7036 (PMC10105308; doi:10.1001/jamanetworkopen.2023.7036)
Supplement: Supplement 2. — Data Sharing Statement [file jamanetwopen-e237036-s002.pdf]

Data Sharing Statement

Chatterjee. Estimated Costs and Outcomes Associated With Use and Nonuse of Medications for Opioid Use Disorder During Incarceration and at Release in Massachusetts. *JAMA Netw Open*. Published April 14, 2023. doi:10.1001/jamanetworkopen.2023.7036

Data
Data available: No

Additional Information
Explanation for why data not available: We are working on making the model available on GitHub but we have not completed that process yet.
